# A CTCF-Binding Element and Histone Deacetylation Cooperatively Maintain Chromatin Loops, Linking to Long-Range Gene Regulation in Cancer Genomes

**DOI:** 10.3389/fonc.2021.821495

**Published:** 2022-01-21

**Authors:** Ran Tang, Yiqun Li, Fang Han, Zhenzhi Li, Xiaoyu Lin, Haoxiu Sun, Xiaoqing Zhang, Qinghua Jiang, Huan Nie, Yu Li

**Affiliations:** School of Life Science and Technology, Harbin Institute of Technology, Harbin, China

**Keywords:** cancer genome, functional DNA element, chromatin loops, CTCF, histone deacetylation

## Abstract

**Background:**

Genes spanning long chromosomal domains are coordinately regulated in human genome, which contribute to global gene dysregulation and carcinogenesis in cancer. It has been noticed that epigenetic modification and chromatin architecture may participate in the regulation process. However, the regulation patterns and functional elements of long-range gene regulation are unclear.

**Methods:**

Based on the clinical transcriptome data from different tumor sets, a novel expressional correlation analysis pipeline was performed to classify the co-regulated regions and subsets of intercorrelated regions. The GLAM2 program was used to predict conserved DNA elements that enriched in regions. Two conserved elements were selected to delete in Ishikawa and HeLa cells by CRISPR-Cas9. SAHA treatment and HDAC knockdown were used to change the histone acetylation status. Using qPCR, MTT, and scratch healing assay, we evaluate the effect on gene expression and cancer cell phenotype. By DNA pull-down and ChIP, the element-binding proteins were testified. 3C and 3D-FISH were performed to depict the alteration in chromatin architecture.

**Results:**

In multiple cancer genomes, we classified subsets of coordinately regulated regions (sub-CRRs) that possibly shared the same regulatory mechanisms and exhibited similar expression patterns. A new conserved DNA element (CRE30) was enriched in sub-CRRs and associated with cancer patient survival. CRE30 could restrict gene regulation in sub-CRRs and affect cancer cell phenotypes. DNA pull-down showed that multiple proteins including CTCF were recruited on the CRE30 locus, and ChIP assay confirmed the CTCF-binding signals. Subsequent results uncovered that as an essential element, CRE30 maintained chromatin loops and mediated a compact chromatin architecture. Moreover, we found that blocking global histone deacetylation induced chromatin loop disruption and CTCF dropping in the region containing CRE30, linked to promoted gene regulation. Additionally, similar effects were observed with CRE30 deletion in another locus of chromosome 8.

**Conclusions:**

Our research clarified a new functional element that recruits CTCF and collaborates with histone deacetylation to maintain high-order chromatin organizations, linking to long-range gene regulation in cancer genomes. The findings highlight a close relationship among conserved DNA element, epigenetic modification, and chromatin architecture in long-range gene regulation process.

## Introduction

In eukaryotic genomes, the co-expression patterns of genes within long chromosomal domains have long been noted (1, 2). These long-range co-expressing regions have been identified in different cancers including bladder (3, 4), colon (5), prostate (6), and breast cancers (7–9). Researchers found that the gene composition of these regions was altered in cancers compared with normal tissue (9). In addition, specific regions were suggested to correlate with different tumor subtypes (8) and tumor grades (4), and some carcinogenesis-associated genes were proved to be regulated directly within the regions (5, 6). These findings strongly support the close correlation between long-range gene regulation and carcinogenesis. Better understanding of the underlying regulatory mechanism of these regions may provide insights into the dysregulation of the cancer genome and aid in identifying new tumor markers and therapeutic strategies.

The regulatory mechanisms of the coordinated gene expression have been explored at both genetic and epigenetic levels. One potential mechanism is the sharing of regulatory elements among genes in these regions, similar to divergent co-expression gene pairs (10) and paralogous gene clusters (11). However, this mechanism cannot completely explain larger-scale and/or non-homologous gene regions (2, 12). Recent advances in nuclear architecture and three-dimensional (3D) genome provided more universal candidate mechanisms and turned the attention to epigenetic level modulation (13, 14). Chromatin, according to specific nuclear locations, is separated into active and inactive regions, which induce long-range gene up- or downregulation on them (15). At the gene-locus level, studies using the Hi-C technique (16) identified the presence of self-interacting chromatin regions called topologically associated domains (TADs) (17) and sub-TADs (18). The CCCTC-binding factor (CTCF) collaborated with the cohesion complex, mediated the interaction between transcription regulatory sequences in different loci (19, 20), and thereby co-regulated gene expression within long-range domains (21, 22). However, the importance of the 3D chromatin structure on long-range gene regulation is still controversial since many genome-wide studies indicated that it had weak impacts on gene expression (9, 14, 23). Additionally, there are few studies on conserved DNA elements that are involved in chromatin architecture maintenance and functional protein recruitment.

In the cancer genome, the connection between long-range gene dysregulation and DNA copy number variants was established very well (24), but a large portion of regions was found to present without CNVs (3, 8). Epigenetic remodeling was considered as another important driver of long-range gene regulation. Long-range epigenetic silencing (LRES) was observed to be associated with coordinately decreased gene expression regions (6) and characterized by increased repressive epigenetic marks such as DNA methylation (5), H3K9 methylation, and H3K27 trimethylation (4, 25). Long-range epigenetic activation (LREA) (26) is associated with overexpressed gene regions and enriched with active chromatin marks like H3K9 acetylation and H3K4 trimethylation (26). Moreover, the 3D chromatin structure was found to participate in these epigenetic regulation processes (27). Chromatin decondensation that is induced by estrogen was shown to promote the expression of genes in a regional epigenetic regulation region (8), and CTCF-derived chromatin loops were found to maintain a long-range epigenetic silenced domain (28). Therefore, epigenetic remodeling is becoming a hotspot in the research of long-range gene co-expression. However, it is still obscure for the most part of regulatory mechanism; the regulation patterns and functional elements of co-expression regions in different tumor types need to be explored.

In this study, using a new analysis pipeline, we explored coordinately regulated regions (CRRs) in eight different tumor sets and classified a subset of CRRs (sub-CRRs) that exhibited a high interregional correlation. We calculated the reoccurred DNA elements in sub-CRRs; one novel DNA element was found to be enriched in sub-CRRs of endometrial cancer and was associated with cancer patient survival. The element was further determined to affect regional gene regulation in sub-CRRs. We clarified that the element contributes to the maintaining of chromatin loops and is associated with regional gene regulation restriction, possibly through CTCF recruiting. We also found that histone acetylation destroyed chromatin loops and disrupted CTCF binding, which was linked to alteration of long-range gene expression in sub-CRRs in cancer genomes. 

## Material and Methods

### RNA-Sequencing Data and Co-Expression Calculation

The expression matrix of genes and the clinical information were collected from The Cancer Genome Atlas Program (TCGA) database; the hg19 UCSC Gene standard track was used for gene location annotation. In each tumor set, every five consecutive genes on the chromosomes were defined as a unit, and the average of the Pearson correlation coefficient of every two genes in a unit was defined as this unit’s average correlation coefficient.

### Detection of CRRs and Sub-CRRs

A unit was defined as a CRR when the correlation coefficients of any two genes in a unit were greater than 0.4 and the adjusted *p* value was less than 0.05. All CRRs with adjacent start positions were merged into one CRR. This definition was more strict than those in previous studies (8, 9), and this strategy leads to relatively short CRRs.

**Figure 1:**
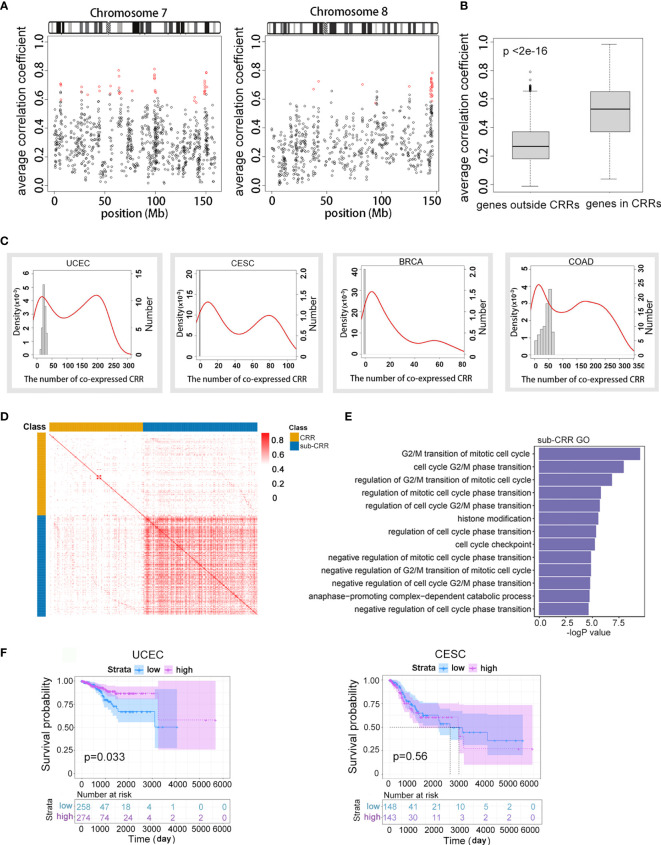
Subset of coordinately regulated regions (Sub-CRRs) exhibited high inter-correlations and were associated with cancer patient survival. **(A)** The average correlation coefficient of each gene with the expression value of four neighbor genes was calculated and mapped on chromosomes 7 and 8 using UCEC data. Red represents CRR regions. **(B)** Boxplots showing the distribution of the average expression correlation coefficient of genes in CRRs and genes outside CRRs using TCGA UCEC data. **(C)** Distribution of CRRs that have an expressional correlation with other CRRs in different cancer genomes. The red density curves correspond to the left ordinate, depicting the proportion of CRR with significant correlation (p < 0.05) with other CRRs; horizontal coordinates indicate the quantities of “other CRRs”. For example, a point (x, y) on the red curve indicates a group of CRRs; each of them has a significant correlation with x (the quantity) other CRRs, and the proportion of such group of CRRs compared with the whole is y × 10^-3^. The histogram corresponds to the right ordinate, depicting the number of CRRs with a significant correlation (p < 0.05) with others under random conditions. **(D)** Heat map of the absolute value of correlations between CRRs. Sub-CRRs are arranged in the blue area while other CRRs are in yellow. Red plots indicate high correlation. **(E)** GO analysis indicated significantly enriched (p < 0.05) items in sub-CRR genes. **(F)** Survival curves of patients with UCEC and CESC classified by expression of genes in sub-CRRs.

For every gene in a CRR, we calculated their average correlation coefficient with any other genes in the same CRR and took it as their scores. A gene with a median score was defined as the representative gene of this CRR and used for calculating correlations between CRRs. The selection of genes with a median correlation coefficient could reduce the problem of outliers caused by regulatory genes. Then, correlation coefficients between every two CRRs were calculated. For each CRR, we counted the number of other CRRs with a high correlation coefficient (>0.4) to it. These counts showed a bimodal distribution (**Figure 1C**). For random situations, the order of all genes in genome was randomized, then CRRs were identified and the interregional correlation coefficient was calculated for each tumor data set. The Gaussian mixed model was used for cluster analysis of this correlation distribution:


$$\text{p}(\text{x})=\sum_{k=1}^{\text{K}}\pi_{\text{k}}\text{N}(\text{x}|\mu_{\text{k}},\Sigma_{\text{k}})$$

where $\text{N}(\text{x}|\mu_{\text{k}},\Sigma_{\text{k}})$ was the kth component in the mixed model and π*_k_* was its weight. The Gaussian mixed model method was used to extract the CRRs in the right peak of the Gaussian distribution, which were defined as the sub-CRRs.

### Detection of Elements

To predict the recurring DNA elements in the sub-CRRs, we used a pipeline based on the MEME suit (29) to predict the elements that were significantly enriched in the sub-CRRs. The full-length DNA sequences of sub-CRRs were obtained from UCSC (http://genome.ucsc.edu/). We used GLAM2 (30) to predict conserved DNA elements that repeatedly appeared in different sub-CRR regions. Using the GLAM2SCAN program, the conserved elements that we found were mapped to the whole genome, then a hypergeometric test was used to find elements that were significantly enriched in the sub-CRRs.

### Statistical Methods and Randomization

All statistical analysis was done with the R project unless otherwise indicated. The t-test was used for differential expression analysis. The Pearson correlation test was used for the correlation test. The hypergeometric test was used for functional enrichment analysis. The Cox regression model was used for survival analysis. The Benjamini–Hochberg method was used for adjusting multiple tests.

The randomization of the units was conducted by randomly selecting genes and four downstream genes. The randomization of the chromosome positions of genes was conducted by random rearrangement of genes on all chromosomes, and the correlations between different CRRs were calculated according to the above methods.

### CRISPR-Cas9 and Deletion Clone Screening

CRISPR-Cas9 was performed as described by Ran et al. (31) with some modifications. Single-guide RNAs (sgRNAs) (**Supplementary Table 1**) were designed on conserved boundaries of the element using the Cas-designer online tool (32) then ligated with linearized CRISPR plasmid using T4 ligase (#EL0011, Thermo Scientific). For deletion of sub-CRR95 CRE30 in the Ishikawa cell line, px330 plasmids were used. 48 h after plasmid transfection, single cells were inoculated and cultured, then genomic DNA was isolated for verification. For other cell lines and loci, sgRNA were cloned into the LentiCRISPR-V2 plasmid; after virus packaging and infection, cells were selected with 3 μg/ml puromycin for 4–5 days. Then genotypes were verified by PCRs using PrimeSTAR^®^ HS DNA Polymerase with GC Buffer (#R044A, Takara Bio.). Primers are listed in **Supplementary Table 1**. For the sub-CRR95 CRE30 locus, 600- and 800-bp bands were obtained for wild-type Ishikawa cells, and the 600-bp band was obtained in wild-type HeLa cells; deletion was confirmed with the presence of the 400-bp band. For the sub-CRR96 CRE30 locus, the 1,200-bp band was obtained for wild-type cells and the 700-bp band was obtained for deletion cells.

### Serum-Starvation and Drug Treatment

Cells were trypsinized and seeded into 12-well plates with medium containing 10% FBS (normal group) or 0.5% FBS (serum-starvation group). After 12 h of incubation, cells were collected. For SAHA treatment, fresh medium was changed with 30 μM SAHA or DMSO and incubated for 24 h before isolation. For SAHA treatment followed by serum starvation, SAHA was added into medium 12 h before starvation; the drug concentration was then maintained for another 12 h during the starvation process. 

### siRNA Knockdown

siRNA oligos are listed in **Supplementary Table 1** and synthesized by GenePharma (GenePharma, Shanghai, China). Cells were transfected with Lipofectamine 2000 (#11668019, Life Technologies), according to the protocol of the manufacturer. After 48 h of culturing, cells were isolated or proceed to serum-starvation experiments. 

### RNA Isolation and Quantitative Real-Time PCR

Total RNA was extracted with TRIzol (#15596018, Invitrogen) and reverse transcript using the PrimeScript RT-PCR Kit (#RR047A, Takara Biotechnology). Real-time PCR was performed by the FastStart Universal SYBR Green Master (#04913914001, Roche) on the Real-Time PCR System (QuantStudio 3, Applied Biosystems). Primers for real-time PCR are described in (**Supplementary Table 1**). β-Actin was used as internal control.

### Cell Proliferation and Scratch Healing Assay

Cell proliferation was measured by 3-(4, 5-dimethylthiazol-2-yl)-2,5-diphenyltetrazolium bromide (MTT) assay. Cells were seeded into 96-well plates; a 12-h culture was proceeded before experiments. MTT was added, and absorbance at 550 nm was measured in time points of 0, 24, 48, and 72 h. At least 6 biological replicates were set for each experiment. For the scratch wound healing assay, cell scratch healing assay was performed as described previously (33). A 3-mm wound was introduced across the diameter of each 12-well plate after a 30-min Mitomycin C treatment. Cell migration was observed by microscopy at 0 and 36 h. Wound width was analyzed objectively using ImageJ.

### DNA Pull-Down

DNA pull-down was based on ref (34). Biotin-labeled primers (**Supplementary Table 1**) were synthetized to amplify the CRE30 sequence using PCR; 300- and 800-bp bands were purified as probes. Biotin-labeled 59-bp random oligos were synthetized as the universal control probe. 10-cm dish Ishikawa cells were harvested after PBS wash and resuspended with Buffer A; after ice incubation, nuclei were isolated by 2,600 g centrifuge and resuspended with Buffer B, and sonication (250 W) was performed for 5 s and incubated on ice for 60 min. Supernatant was collected as the nuclear lysis solution after being centrifuged. 100-μl probes (100 nM) and 200 μl nuclear lysis solution were added into a 700-μl binding buffer and incubated for 30 min at room temperature. 50-μl streptavidin-agarose beads (20361, Pierce™) were added and incubated for 60 min on a rocking platform at room temperature. Beads were washed with binding buffer, then incubated at 95°C for 5 min. Samples were centrifuged, and supernatant was collected for further detection. 300-bp probe samples were used for SDS-PAGE and silver staining to confirm the efficiency; samples were sent out (Wuhan GeneCreate Biological Engineering Co.) for mass spectrum analysis.

### ChIP Assay

ChIP assay was performed according to a previous description (35) with some modifications. Cells were fixed by adding formaldehyde and incubated at 37°C for 2 min (H3K27ac ChIP) or 20 min (CTCF, Rad21ChIP). Cross-linking was stopped with glycine. Cells were harvested and incubated in 1 ml cellular lysis buffer and centrifuged, and then the pellet was resuspended in 300 μl nuclear lysis buffer. Sonication was performed (250 W, 20-s run, 60-s pause, 4 times). Supernatant DNA was collected, and the concentration was detected on NanoDrop. 20 μg chromatin was used for each ChIP sample, and 30-μl protein A/G magnetic beads (HY-K0202, MCE) were added into a chromatin solution; 5 μl IgG (ab172730, Abcam) or 5 μl CTCF (D31H2, Cell Signaling) or 1.5 μl H3K27ac (ab177178, Abcam) or 3.4 μl Rad21 (EPR22506-15, Abcam) antibody was added then incubated overnight. The beads were washed twice with dilution IP buffer, once with TSE buffer, once with LiCl buffer, and twice with TE buffer. A binding chromatin was eluted with elution buffer and digested with RNase and proteinase K. Beads were removed, and the chromatin was incubated at 65°C for 4 h for decrosslinking. The DNA segments were purified by a PCR purification kit (#K0702, Thermo Scientific). Primers of ChIP-qPCR (**Supplementary Table 1**) were designed according to the peak location of ChIP-seq data; all loci were tested in triplicate. Primers for the CRE30 locus were designed closely beside boundaries, and qPCR primers on the upstream boundary and PCR primers on the downstream boundary of sub-CRR95 CRE30 were designed. For the sub-CRR96 locus, because of the existence of primer dimers, only PCR primers were designed on the upstream boundary (**Supplementary Table 1**). Sonication parameters for the CRE30 locus testing samples were adjusted to generate slightly longer (about 500 bp) chromatin segments: 150 W, 15-s run, 60-s pause, 4 times. Each experiment was performed in biological duplicate.

### 3C-qPCR

3C-qPCR experiments were performed according to the Nature Protocol (36) with some modifications. Formaldehyde was used for cell fixation and quenched with glycine. Cells were harvested and resuspended with lysis buffer. The nucleus was extracted by centrifugation and then resuspended with 1.2x CutSmart Buffer (#B7204S, New England Biolabs); SDS and Triton X-100 were added followed by 1-h incubation, respectively. Hind Ш enzyme (#R3104L, New England Biolabs) was added for genomic DNA digestion, and reaction was stopped by SDS. The samples were moved into T4 ligase buffer (#B0202S, New England Biolabs), and ligation was proceeded by adding 800 U T4 DNA ligase (#M0202V New England Biolabs), and at 16°C a 4-h incubation followed, with an additional 1-h incubation at room temperature, 65°C overnight for decrosslinking. Then, the DNA was purified with phenol: chloroform: isoamyl alcohol. DNA was precipitated with ethanol and washed with 70% ethanol, then dissolved in water. Samples were assayed by qPCR in triplicate using a TaqMan qPCR mixture (#4444557, Applied Biosystems). Bait and test primers were designed according to a genomic Hind Ш restriction map and CTCF ChIP-seq peak location; we tested all Hind Ш fragments which contain a CTCF-binding peak and gene promoters in the sub-CRR95 region. In order to control for the amplification efficiency of primers, DNA fragments (longer than 1 kbp) that contain an up- and downstream sequence around Hind Ш digestions were cloned and purified for each tested restriction site and mixed together with equal copy numbers; equal efficiency for primers was confirmed with positive interaction. A FAM-labeled TaqMan probe (**Supplementary Table 1**) was designed on the amplification region of the bait segment. 

### 3D-FISH

Customization FISH probes and a hybridization buffer were purchased from Empire Genomics LLC. (USA); a probe mapped to the upstream boundary of sub-CRR95 was labeled with 5-fluorescein and the downstream boundary probe labeled with 5-ROX. The experiments were performed according to the protocol of the manufacturer. Cells were seeded onto a round coverslip; after 24 h of culturing, cells were fixed with 4% PFA, permeabilized with 0.5% Triton X-100, then immerged in 20% glycerine; 3× freeze/thawing cycles were performed in liquid nitrogen. Coverslips were incubated with 0.1 M HCl and then 0.5% Triton X-100 with twice PBS washes before and after each step. Before hybridization, coverslips were immerged in 50% formamide/2xSSC for at least 30 min, then incubated in each of 2xSSC and 70%, 85%, and 100% ethanol sequentially. Probe solutions (3.2 μl hybridization buffer, 0.4 μl green probe, and 0.4 μl red probe) were added then sealed with rubber cement. Slides were heated at 74°C for 4 min and incubated at 37°C for 24 h. Coverslips were washed in WS buffer1 (0.3% NP-40, 0.4xSSC) at 73°C for 2 min then transferred into WS buffer2 (0.1% NP-40, 2xSSC) for 1 min at room temperature. Coverslips were dried in the dark and sealed with anti-fade regent containing DAPI (#P36941, Qiagen). Images of 3D-FISH were captured using a fluorescence confocal microscope (LSM880, Zeiss), then the 3D distance between red and green loci was measured by ZEN software. 90–120 loci were measured for each sample. 

## Results

### Subset of CRRs Exhibited a High Interregional Correlation in Cancer Genomes and Were Associated With Cancer Patient Survival

To explore the regulation patterns and functional elements of long-range gene regulation in cancer genomes, we first assessed coordinate expression patterns in eight cancers from the TCGA database (**Table 1**). A relatively strict method (see *Methods*) was used to calculate the expression correlation of adjacent genes in genomes; the coordinately regulated regions (CRRs) were identified as regions in which the absolute value of the Pearson correlation coefficient between any pair of genes was greater than 0.4 (adjusted p < 0.05). The characteristics of CRRs in all eight tumor datasets are shown in **Table 1**. Gene numbers of each CRR were from 5 to 40, the spanning distance was from 4 kb to 5.69 Mb, and the median distance was distributed between 0.22 and 0.3 Mb. In addition, the numbers and distributions of total CRRs in different tumors showed a wide range (from 115 to 517). While the intersections of CRRs between different tumors were less than 25% (data not shown), which meant a high tumor heterogeneity in the regional co-expression patterns. The distribution of CRRs on chromosomes is shown in **Figure 1A** and **Figures S1A, B**. Using endometrial cancer as an example, we observed that the average correlation coefficient of any adjacent five genes in CRRs was significantly higher than other random adjacent regions in the genome (p < 2e-16, **Figure 1B**). These results illustrated that our method could correctly identify DNA regions with a high expression correlation.

**Table 1:** Characteristics of CRRs obtained from different tumor datasets.

Tumor dataset *	Quantity of CRRs	Mean of gene quantity	Range of gene quantity	Mean of spanning distance (Mb)	Median of spanning distance (Mb)	Range of spanning distance (Mb)
UCEC	369	6.3	5–34	0.39	0.28	0.004–3.49
CESC	183	5.8	5–15	0.33	0.29	0.020–1.75
BRCA	214	6.2	5–23	0.29	0.22	0.003–1.68
COAD	517	6.3	5–16	0.43	0.3	0.004–5.69
HNSC	150	6	5–16	0.33	0.25	0.010–2.03
KIRC	115	5.9	5–13	0.33	0.26	0.008–2.13
KIRP	196	6	5–25	0.39	0.28	0.010–3.46
SKCM	175	6.5	5–40	0.35	0.23	0.020–1.36

*UCEC, uterine corpus endometrial carcinoma; CESC, cervical squamous cell carcinoma and endocervical adenocarcinoma; BRCA, breast invasive carcinoma; COAD, colon adenocarcinoma; HNSC, head and neck squamous cell carcinoma; KIRC, kidney renal clear cell carcinoma; KIRP, kidney renal papillary cell carcinoma; SKCM, skin cutaneous melanoma.

Next, interregional correlations between different CRRs were examined to look for CRRs exhibited similar regulation patterns. For each CRR, we calculated the quantity of other CRRs with a high correlation coefficient (>0.4) with it, and the distributions were represented by density curves (**Figure 1C** and **Figure S2A**). Interestingly, the density curves presented a typical bimodal distribution in many cancers. The peak on the left side overlapped the peak generated by random situations (column chart, see *Methods*), and these suggested a randomly occurred correlation and were excluded from subsequent analysis. The right-side peak consisted of CRRs that have more correlated partners than random. This result shows that the correlations between some CRRs were significantly higher (p < 2e-16) than those in random cases. These CRRs were defined as sub-CRRs and were obtained using a clustering method based on the Gaussian mixture model (**Supplementary Table 2**). To further evaluate the intra-correlations within sub-CRRs, a heat map of the absolute value of interregional correlation coefficients of CRRs of endometrial cancer was clustered (**Figure 1D**). A significantly higher correlation was observed among sub-CRRs than that of other CRRs (t test, p < 2e-16) as well as between sub-CRRs and the other CRRs. The high correlations among these co-regulated regions suggested that sub-CRRs might potentially share same regulatory mechanisms.

We further observed that many genes in sub-CRRs were closely related to cancer characteristics. Gene Ontology (GO) analysis of genes contained in sub-CRRs revealed functions related to the cell cycle and proliferation (**Figure 1E**). According to the gene expression pattern of sub-CRRs, TCGA clinical cases were classified into different subtypes using the DBSCAN density clustering method, and survival analysis was then performed. The results showed that survival rates have significant difference between subtypes in several cancers (**Figure 1F** and **Figure S2B**).

Together, our results identified co-regulated regions: CRRs in different tumor sets, and classified sub-CRRs exhibiting high interregional correlations with each other. Regional regulation mechanisms might be shared among sub-CRRs; therefore, we next analyzed the sub-CRRs and searched for functional DNA elements that are involved in the co-regulation mechanism. 

### A Conserved DNA Element Containing Repetitive Motifs Was Enriched in Sub-CRRs

**Figure 2:**
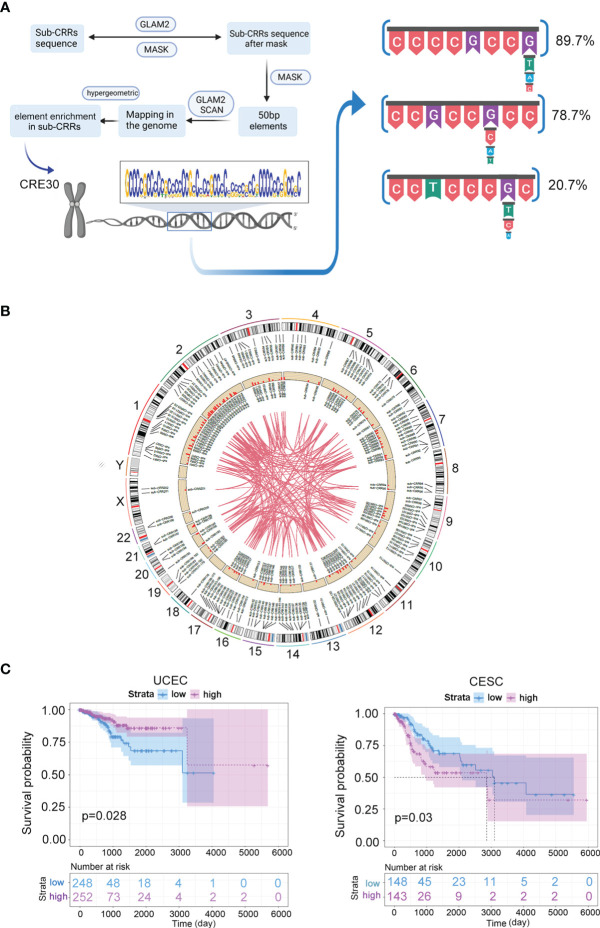
CRE30 enriched in sub-CRRs and associated with clinical case survival. **(A)** Schematic presentation of the conserved DNA element prediction pipeline performed in this study based on the MEME program. Representative segments of CRE30 were obtained through searching of matched sequences in the reference genome (hg19); the repeat motifs that frequently appeared are shown on the right side. **(B)** The circus plot illustrates the locus information of sub-CRRs and CRE30s on chromosomes in the UCEC genome. The outer circle is the location of sub-CRRs on chromosomes, and the inside circle is the location of sub-CRRs containing CRE30. The histogram shows the proportion of other CRRs that have strong correlations (r > 0.6) with the given sub-CRR; the maximum ordinate is 1. The curves in the center connect sub-CRRs with strong correlations (r > 0.6). **(C)** Survival curves of UCEC and CESC clinical groups classified by genes in sub-CRRs with CRE30.

To identify the functional elements enriched in sub-CRRs, a pipeline based on the MEME program (29) was performed (see *Methods*). Seventy conserved DNA sequences with a high frequency in sub-CRRs were obtained in endometrial cancer. We found that the No. 30 DNA sequence was significantly enriched in the sub-CRRs compared with whole genome (hypergeometric test, p < 1e-7), and we named this sequence as core regulation element 30 (CRE30). CRE30 contains mostly G and C bases and exhibits an extraordinarily high GC content (**Figure 2A**). Through searching matched sequences in the reference genome (hg19) with MEME, DNA segments in the whole genome containing CRE30 were obtained. These segments were constructed by a series of repetitive motifs, which mainly include ccccgccg(t/a/c), ccgccg(c/a/t)ccg, and cctcccg(t/c/a)c with the frequency of 89.7%, 78.7%, and 20.7%, respectively. Among these repetitive motifs, there were non-uniform interval sequences with various lengths and sequence contents. Then, we analyzed characteristics of sub-CRRs containing CRE30 and found that they were widely distributed on all chromosomes in endometrial cancer (**Figure 2B**). A large amount of correlation coefficients among them was higher than 0.6. Enrichment analysis of CRE30 was also performed in cervical carcinoma, and CRE30 was also significantly enriched in the sub-CRRs in the cervical cancer genome (hypergeometric test, p < 2e-7).

Based on the clinical and gene expression data from TCGA datasets, we used gene expression data in sub-CRRs containing CRE30s to divide the clinical cases into two subtypes. Survival analysis based on Cox regression was performed. As shown in **Figure 2C** and **Figure S3**, significant differences in survival were observed in UCEC, CESC, BRCA, COAD, and KIRC. Notably, the survival rates in CESC, COAD, and KIRC were significantly different between groups classified by sub-CRRs with CRE30, while no difference was observed when using sub-CRRs (**Figure 1F** and **Figure S2B**). The results suggested that sub-CRRs with CRE30 might provide a more precise range, which included genes that participate in the processes of carcinogenesis and malignant progression. 

### Deletion of CRE30 in Sub-CRR95 Affects Long-Range Gene Regulation and Cancer Cell Phenotype

**Figure 3:**
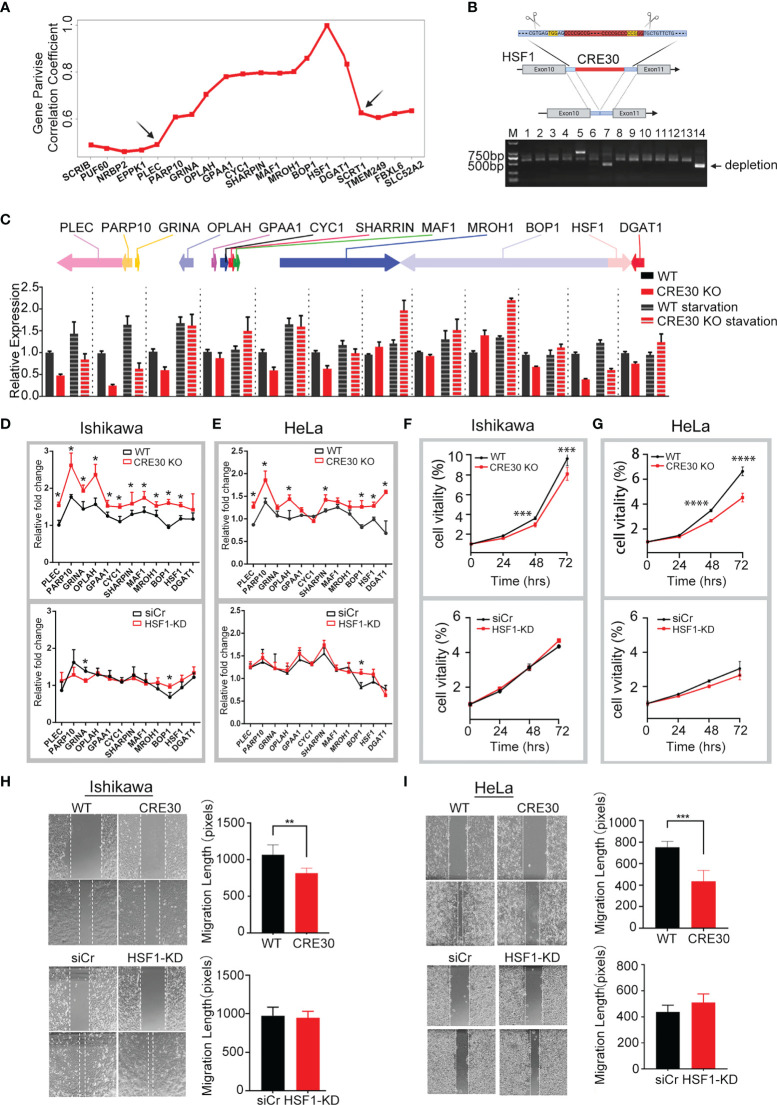
CRE30 deletion in sub-CRR95 affected gene regulation and tumor cell phenotype. **(A)** Distribution of expression correlation coefficients among genes around sub-CRR95. Arrows indicate the boundary sites. **(B)** Upper panel, schematic showing CRE30 location and CRISPR deletion process in the HSF1 gene region; the red rectangle indicates the CRE30 locus, and PAM sequences used for CRISPR are shown in yellow. Lower panel, PCR bands for both wild-type and deletion clones. PCR of wild-type clones results in two bands, while only one band (less than 500 bp) was obtained from the double deletion clone. **(C)** qPCR analysis for sub-CRR95 gene expression following deletion of CRE30 in cells with regular or serum starvation culture. Black columns indicate wild-type cells, and red columns indicate CRE30 KO cells (n = 3). **(D, E)** Relative fold change of sub-CRR95 gene expression under serum-starvation stress in Ishikawa **(D)** and HeLa **(E)** cells. Expression ratio of serum starvation to regular condition is shown to reflect gene regulation under stress. Upper panel shows differences between wild-type (black) and CRE30 KO cells (red) cells; lower panel compares HSF1 knockdown (red) with control (black) groups in wild-type cells (n = 3). Asterisks indicate the significant differences. **(F, G)** Cell proliferation in response to CRE30 deletion (upper panel) and HSF1 knockdown (lower panel) as determined by MTT assays in Ishikawa **(F)** and HeLa cells **(G)** (n ≥ 6). **(H, I)** Wound healing experiments were used to evaluate cell migration in response to CRE30 deletion (upper panel) and HSF1 knockdown (lower panel) in Ishikawa **(H)** and HeLa **(I)** cells (n ≥ 4). T-tests were performed, and significant differences were indicated by * (p < 0.05), ** (p < 0.01), *** (p < 0.001) and **** (p < 0.0001).

To assess whether loss of CRE30 resulted in alteration of gene expression in sub-CRRs, we conducted the CRISPR/Cas9-based sequence deletion of CRE30 insub-CRR95 (**Supplementary Table 2**), which is composed of 12 genes located at 8q24.3. All genes in the region had a very high average correlation coefficient with low value boundaries on both sides (**Figure 3A**). In sub-CRR95, CRE30 is fully contained in the 10th intron of the HSF1 gene (**Figure 3B** and **Figure S4A**). Based on the reference genome, the CRE30 segment is 190 bp long. Sequence analysis by Tandem Repeats Database (37) revealed that the segment mainly contains the core repeat unit “CCCCGCC(G/T)” which was also frequently detected in CRE30 in other genomic regions (shown in **Figure 2A**). Some short sequences such as “CG” or “CCGCCG” were found between repeat units, and the copy number of repeat units exhibited polymorphism among different cell lines or sister chromatids (**Supplementary Table 3**). The CRE30 locus in HSF1 in Ishikawa cells was also found as heterozygous with 200- and 400-bp length (**Supplementary Table 3**). Thus, both segments were deleted by CRISPR-Cas9 (**Figure 3B** and **Figure S4A**); two single clones (CRE30 KO cells) were selected for subsequent experiments. FISH was performed to make sure there is no chromosomal abnormality in KO cells (**Figure S4B**), and sequences of HSF1 mRNA were explored to confirm that element deletion did not lead to incorrect splicing or gene coding errors (**Figure S4C**).

We then compared the gene expression change between wild-type and CRE30 deletion cells. In the region of sub-CRR95, the expressions of two genes were increased and the expressions of six genes were decreased in cells cultured in regular medium, indicating a regional dysregulation after CRE30 deletion (**Figure 3C**). Transcriptional regulation can respond to environmental changes in a relatively short period of time, and therefore, we used 12-h serum starvation as a short-term stress to examine differences in the dynamic gene regulation process. As shown in **Figure 3C**, all 12 genes in the KO group were upregulated remarkably under starvation stress. In comparison, in the WT group, only six genes were upregulated and others showed only a modest change. In comparing the upregulation fold change after starvation stress in each group (**Figure 3D** upper panel), we observed that the transcriptional upregulation scope of genes in the KO group was significantly higher than the wild type, and this appeared in almost all genes. The results suggested that larger stress-induced expressional alterations appeared after CRE30 deletion.

Considering that the HSF1 expression decreased markedly after CRE30 deletion (**Figure 3C**), HSF1 was knocked down in wild-type cells to determine whether the alteration of gene regulation in this region was induced from HSF1 expressional change. qPCR results confirmed that HSF1 RNA levels in knockdown cells were lower than those in CRE30 KO cells (**Figure S4D**), and the expressional fold change of the region genes showed no difference (**Figure 3D** lower panel). Increased changes in gene expression were from CRE30 deletion but not HSF1 knockdown and demonstrated that CRE30 was the cause of long-range gene expressional change in sub-CRR95.

Meanwhile, sub-CRR95 was also identified in the cervical carcinoma genome. To explore the effect of CRE30 in other tumor types, the CRE30 segment was deleted in the HeLa cell line by the Lenti CRISPR V2 screening system (**Figure S4E**). Similar to Ishikawa cells, HeLa cells deleted for CRE30 showed a larger upregulation of gene expression under starvation (**Figure 3E** upper panel, **Figure S4F**), but no difference was observed in cells with HSF1 knockdown (**Figure 3E** lower panel, **Figure S4G**). We next examined cancer cell phenotypes in CRE30 deletion and wild-type cells. MTT and wound healing experiments showed that the CRE KO cells exhibited reduced proliferation and migration in both cell lines, while the HSF1 knockdown groups showed no differences (**Figures 3F–I**). This suggests that the regulation process mediated by CRE30 had significant implications on cancer cell phenotypes.

### CRE30 Recruits CTCF and Maintains Chromatin Loops in Sub-CRR95, Contributing to Compact Chromatin Architecture

**Figure 4:**
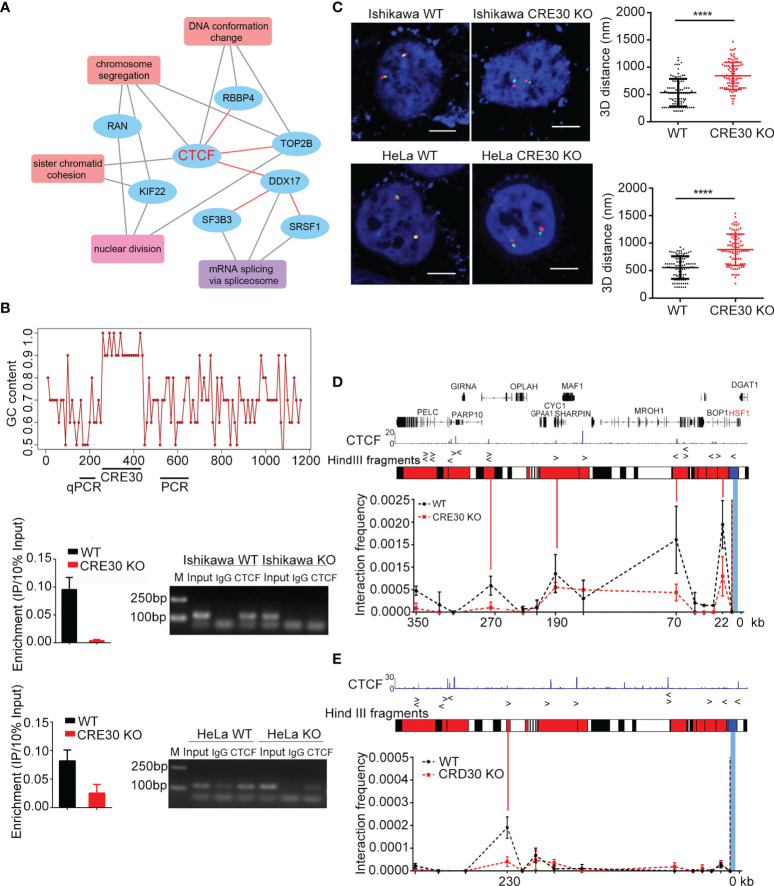
CTCF binding to the CRE30 locus acts to maintain chromatin architecture. **(A)** Enriched GO terms for CRE30-binding proteins. Black lines indicate the proteins that are included in each item; red lines indicated interaction events between protein candidates. **(B)** Upper panel, line chart represents genomic GC content (calculated for each of 10 bases) around the sub-CRR95 CRE30 locus. The GC content within the element region was higher than 90%. Horizontal bars on the bottom indicate where ChIP-qPCR and ChIP-PCR primers were designed. Middle (Ishikawa cells) and lower panel (HeLa cells), qPCR (left) and PCR (right) were performed to detect CTCF binding in wild-type and CRE30 deletion cells (n = 3). **(C)** Left, representative 3D-FISH images of Ishikawa cells (top) and HeLa cells (bottom) before and after CRE30 deletion. Boundary loci of sub-CRR (red and green) and DAPI (blue) are shown. Scale bars indicate 5 μm. Right, scatter diagrams show 3D distance between adjacent red and green spots, suggesting a larger distance after CRE30 deletion. Asterisks indicate the significant differences; approximately 90–120 loci were counted for each group. **(D, E)** 3C-qPCR at the sub-CRR95 region. CTCF binding peaks are shown on the top according to previous ChIP-seq data in Ishikawa (ENCFF961BQG, ENCODE) and HeLa cells (38). Middle, bars illustrate Hind Ш sites across the region. Based on the CTCF-binding location, bait fragment (blue) and interacting fragments (red) were designed and tested. Line chart at the bottom showed looping intensity in Ishikawa **(D)** and HeLa **(E)** cells. Bait fragment is indicated by the blue bar, and interacting fragments are shown by red solid lines. Multiple interaction peaks were detected in wild-type samples (black dotted lines), which declined in CRE30-deletion samples (red dotted lines) (n ≥ 2). **** indicates where t-test, p < 0.0001.

Then we examined whether specific proteins were recruited to the CRE30 locus and participated in the regulation process. We performed DNA pull-down experiments to isolate CRE30-binding proteins and identified the proteins by mass spectrometry (**Figure S5A**). According to heterozygous DNA length on the Ishikawa CRE30 locus, two DNA probes (300 and 800 bp long) were used, and 25 binding proteins were determined from the intersection (**Supplementary Table 4**). Protein candidates were searched in protein–protein interaction networks and enriched for 13 GO items (**Supplementary Table 5**). We found that “DNA conformation change” (GO:0071103) was significantly enriched (p adjust < 0.001) (**Figure 4A**), implying a potential role of CRE30-binding proteins in maintaining chromatin architecture. Within this item, CTCF which had high interacting frequency with other proteins attracted our attention.

CTCF-ChIP experiments were then performed to confirm the recruitment reversely. According to the ultra-high GC content, it was failed to find an amplifiable DNA segment within the body of CRE30. Therefore, we detected binding signals on two boundaries of the CRE30 segment using qPCR (left boundary) and PCR (right boundary) (**Figure 4B**). As the results of both methods, clear CTCF-binding signals were observed in Ishikawa and HeLa cells, and the signals decreased significantly after CRE30 deletion (**Figure 4B**). To better control the ChIP signals on the CRE30 locus, we assayed the locus 700 bp upstream and 750 bp downstream of boundaries, and no obvious signals were obtained (**Figure S5B**). We also detected other CTCF-binding sites in sub-CRR95 and found no obvious change with CRE30 deletion (**Figure S5C**). These results confirmed CTCF binding on the CRE30 segment in sub-CRR95. We also knocked down CTCF by siRNAs and examined gene expression changes under stress but did not detect widespread changes in expression (**Figures S5D, E**). Further ChIP analysis showed that CTCF knockdown did not affect the amount of CTCF binding to the CRE30, which indicated a “persistent” binding event (28) on CRE30 (**Figure S5F**).

Considering the well-established relationship between CTCF and high-order chromatin organization (19, 21), we used three-dimensional DNA fluorescent *in situ* hybridization (3D-FISH) to examine whether loss of CTCF binding that is induced by CRE30 deletion could affect the chromatin architecture around sub-CRR95. Green and red fluorescence probes were designed on the two boundaries of sub-CRR95 (**Figure S5G**), and the 3D distance between green and red loci was calculated (**Figure 4C** and **Figure S5H**). We observed that the distance between the two boundaries increased significantly after CRE30 deletion in both Ishikawa and HeLa cells, which indicated that the chromatin structure in the sub-CRR95 region was altered from a relatively compact shape to a loose structure with CRE30 deletion. These results suggested that CRE30 plays an essential role in maintaining a compact chromatin architecture of sub-CRR95.

To gain more insights into CRE30 and CTCF-derived chromatin loop(s), we performed chromosome conformation capture (3C) experiments. A bait primer was designed in the Hind Ш restrict fragment which contains the CRE30 locus, and restriction fragments that cover CTCF-binding peaks were designed as the detection targets (**Figure 4D**). In wild-type Ishikawa cells, we identified at least four interaction peaks derived from the CRE30 locus. These peaks were located approximately 350, 270, 190, 70, and 22 kb upstream of the CRE30 locus, widely distributed in the whole sub-CRR range. In CRE30 KO cells, three of the four peaks were markedly reduced, indicating the disruption of chromatin loops on the locus (**Figure 4D**). A similar trend was observed in HeLa cells; an interaction peak was detected in the wild-type cells, close to the upstream boundary of sub-CRR95, and this was decreased significantly in CRE30 KO cells (**Figure 4E**). We speculate that CRE30 bound by CTCF participated in maintaining these loops. According to the well-established model that the cohesion complex co-binds at the CTCF site and contributes to the maintaining of chromatin loops (21, 39), we also assessed if cohesion was also recruited on the CRE30 locus. ChIP-qPCR and ChIP-PCR were performed for the cohesion subunit Rad21 at CRE30 boundaries (**Figure S5I**), and results demonstrated that Rad21 also bound on the locus and the binding was reduced with CRE30 deletion.

Together, these results indicated that through recruiting CTCF and maintaining chromatin loops in sub-CRR95, CRE30 acts as an essential element for holding compact chromatin architecture. CRE30 deletion led to loop disruption and chromatin decondensation, which further linked to changes in gene regulation patterns within the region. 

### Blocking Histone Deacetylation Induced CTCF Detaching and Chromatin Loop Disruption That Further Affected Long-Range Gene Expression

**Figure 5:**
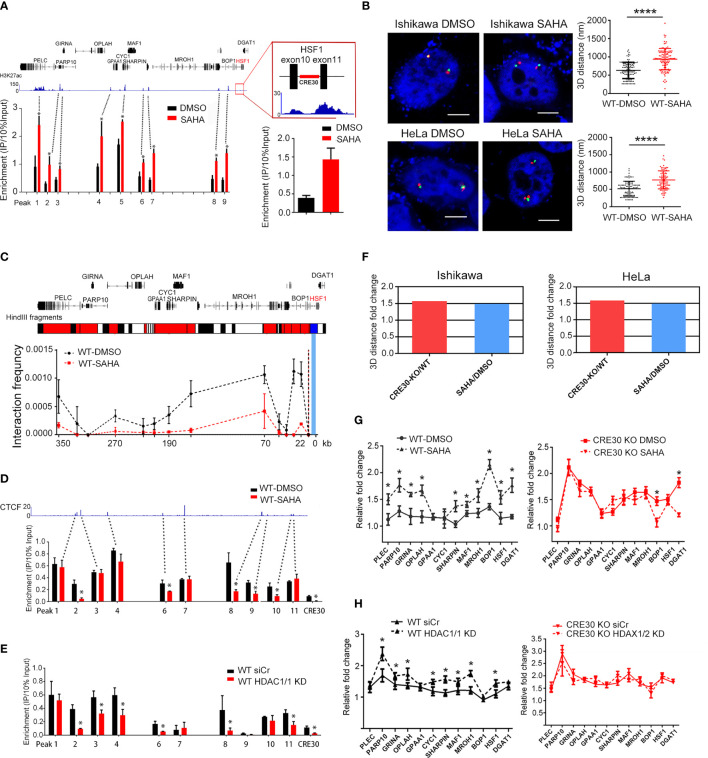
Histone acetylation affects chromatin architecture and regional gene regulation in sub-CRR95. **(A)** H3K27ac ChIP-qPCR experiments were performed in Ishikawa cells following SAHA treatment. Primers were designed on modification peak loci obtained from ChIP-seq data in uterus (ENCFF405MES, ENCODE). Peak number corresponds to primers listed in **Supplementary Table 1**. A significant increase of H3K27ac levels was detected in the SAHA treatment group (red bars) compared with the DMSO treatment group (black bars) (n = 3). Right panel zoom in the CRE30 locus. **(B)** Representative 3D-FISH images of Ishikawa cells (upper panel) and HeLa cells (lower panel) with DMSO or SAHA treatment. Boundary loci of sub-CRR (red and green) and DAPI (blue) are shown. Scale bars indicate 5 μm. Scatter diagrams on the right show the 3D distance between adjacent red and green spots, suggesting a significant increase after SAHA treatment in both cell lines. Approximately 90–120 loci were counted for each group. **(C)** Analysis by 3C-qPCR in Ishikawa cells at the sub-CRR95 region. The Hind Ш fragment bars on the top illustrate bait fragments (blue) and tested interaction fragments (red). Interaction peaks were detected in the DSMO group (black dotted lines), which decreased with SAHA treatment (red dotted lines) (n ≥ 2). **(D, E)** ChIP-qPCR assay showed that CTCF binding decreased widely following both SAHA treatment **(D)** and HDAC1/2 knockdown **(E)** in Ishikawa wild-type cells (n = 3). Primers designed on CTCF peaks according to ChIP-seq data in Ishikawa (ENCFF961BQG, ENCODE); peak number corresponds to primers listed in **Supplementary Table 1**. **(F)** Based on the 3D distance that is obtained in the same batch of 3D-FISH experiments; fold changes of mean 3D distance were calculated in the CRE30 KO group (CRE30 deletion/wild-type) and SAHA treatment group (SAHA/DMSO). Results in both Ishikawa and HeLa cells are displayed. **(G, H)** qPCR experiments were performed for wild-type Ishikawa cells following SAHA treatment **(G)** and HDAC1/2 knockdown **(H)**. Gene upregulation increased in the wild-type group (left panel) in both conditions, with little or modest change in CRE30 deletion cells (right panel) (n ≥ 2). Asterisks indicate the significant differences. T-tests were performed, and significant differences were indicated by * (p < 0.05) and **** (p < 0.0001).

We further wondered if CRE30-mediated chromatin loops were modulated in cancer genomes. Previous studies suggested that direct epigenetic modifications, such as histone acetylation on CTCF-binding sites and chromatin loop boundaries, impact the maintenance of chromatin loops (40–43). We used the histone deacetylase inhibitor SAHA to treat cell lines, and chromatin architecture and loops were detected to examine potential changes with increased histone acetylation. Firstly, H3K27ac ChIP-qPCR was performed to evaluate acetylation status; the peaks from uterus ChIP-seq data (ENCFF405MES, ENCODE) were assayed. We found that the modification signals increased markedly in response to SAHA treatment (**Figure 5A** left) as well as the CRE30 locus (**Figure 5A** right), which suggested a higher histone acetylation level in the sub-CRR95 region induced by SAHA.

To assess the effects of histone acetylation on chromatin architecture, we performed 3D-FISH experiments on Ishikawa and HeLa cells, and the results showed a significant increase of the 3D distance between sub-CRR95 boundaries after SAHA treatment compared with controls (**Figure 5B** and **Figure S6A**). These results reflected a relative loose chromatin conformation induced by histone acetylation that was similar with CRE30 deletion. Next, to confirm the effect on CRE30-derived chromatin loops, 3C experiments were performed on both cell lines (**Figure 5C** and **Figure S6B**, upper). Again, similar to the results with CRE30 deletion, the interaction frequency of all peaks was significantly decreased. These results confirmed that histone acetylation induced chromatin loop disruption in sub-CRR95.

To explore the possible driving force of chromatin architecture changes, we next conducted CTCF ChIP. In Ishikawa cells, the CTCF-binding signals on many tested loci including CRE30 were decreased under SAHA treatment (**Figure 5D**). Similar results were obtained in SAHA-treated HeLa cells (**Figure S6B** lower). To further confirm this observation, we knocked down both HDAC1 and HDAC2 histone deacetylases by siRNAs (**Figure S6C**) and evaluated CTCF binding. Similar to results from enzymatic inhibition, genetic knockdown of the histone deacetylases also caused a decrease in CTCF binding to many loci including CRE30 (**Figure 5E**). Then, we assessed Rad21-binding signals in the region by ChIP-qPCR (**Figure S6D**) and found that after SAHA treatment, Rad21 binding was also reduced widely. These observations suggested that histone acetylation influenced CTCF binding in many loci of sub-CRR95 including CRE30.

According to the 3D distance data that were obtained from the same batch of 3D-FISH experiments, we compared the fold change of the 3D distance with CRE30 deletion and SAHA treatment. The decrease in fold change of boundaries proximity induced by SAHA treatment was mostly identical to that with CRE30 deletion (**Figure 5F**). Considering that SAHA induced CTCF detaching (**Figure 5D** and5 **Figure S6B** lower), this result indicated that even in the presence of CRE30 (wild-type cells), the decrease in CTCF binding had a similar effect on chromatin architecture compared with element deletion. This suggests that CRE30 contributes to chromatin loops and compact chromatin architecture possibly through CTCF recruitment.

To verify if chromatin loop disruption induced by histone acetylation was concomitant with gene expression alteration, we evaluated fold changes of gene expression under serum-starvation conditions. In Ishikawa cells, gene upregulation in wild-type cells was increased by both SAHA treatment (**Figure 5G** left) and HDAC1/2 knockdown (**Figure 5H** left). These results were also confirmed in HeLa cells (**Figure S6E**). These expressional changes in wild-type cells confirmed the close correlation between compact chromatin architecture and restricted gene regulation patterns in sub-CRR95 and suggested a role for histone acetylation in modulating sub-CRR gene expression. Moreover, upregulation after histone deacetylase manipulation in wild-type cells reached a similar level as CRE30 deletion with DMSO, while the same treatment could not further promote gene upregulation in CRE30 KO cells (**Figure 5G** right, **Figure 5H** right, and **Figure S6F**). Based on the same effect on chromatin architecture induced by CRE30 deletion and histone acetylation, these observations further support the functional roles of chromatin architecture on the dynamic gene regulation process in sub-CRR95.

These findings show that histone acetylation influences CTCF binding and disrupts chromatin loops that are mediated by CRE30, which further link to gene expressional changes in sub-CRR95. Moreover, the results further support the important roles of CTCF in CRE30-maintained chromatin architecture.

### CRE30 Exhibits Similar Effects on Other Sub-CRR Loci of Cancer Genomes

**Figure 6:**
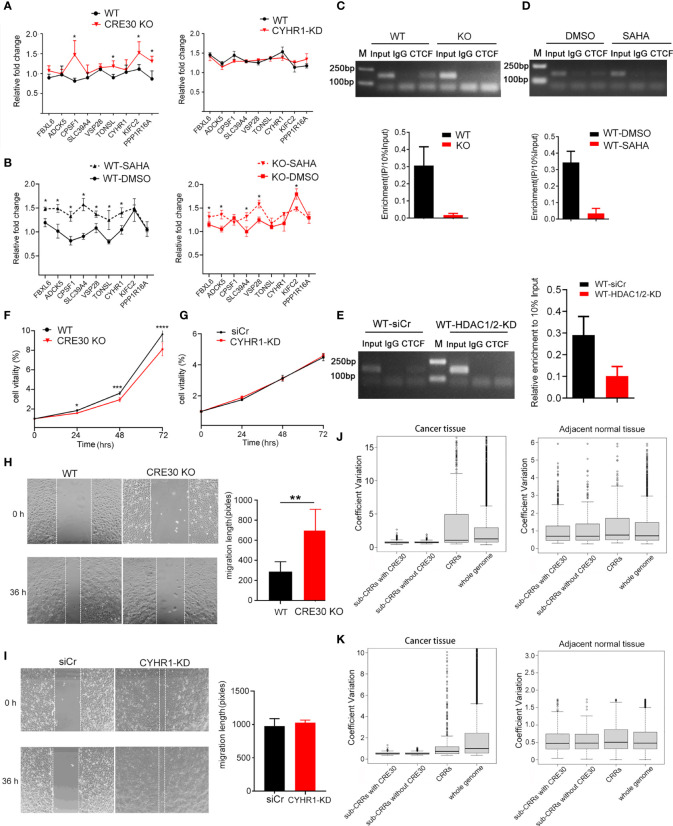
CRE30 exhibited similar effects in other sub-CRRs in cancer genomes. **(A)** In the sub-CRR96 region, qPCR assay showed an increase of gene upregulation scope in CRE30-deleted Ishikawa cells (left panel), while CYHR1 knockdown only had modest changes (right panel) (n ≥ 3). **(B)** In the sub-CRR96 region, treatment with SAHA induced significantly larger gene regulation in wild-type Ishikawa cells (black dotted line, left panel), while the gene regulation scope showed a smaller increase in CRE30 deletion cells (red dotted line, right panel), n = 3. **(C)** CTCF ChIP-PCR confirmed CTCF binding at the CRE30 locus in sub-CRR96, and binding decreased with CRE30 deletion (n = 3). Asterisks indicate the significant differences. **(D, E)** ChIP-PCR results showed a decline of CTCF binding at the sub-CRR96 CRE30 locus in response to SAHA treatment **(D)** and HDAC1/2 knockdown **(E)** in Ishikawa cells (n ≥ 2). **(F, G)** MTT assays for Ishikawa cells indicated decreased cell proliferation in sub-CRR96 CRE30 deletion cells **(F)**, but not in CYHR1 knockdown cells **(G)** (n ≥ 6). **(H, I)** Wound healing assay for Ishikawa cells suggested CRE30 deletion **(H)** at the sub-CRR96 locus induced a significant decline in cell migration, while no difference was observed with CYHR1 knocking down **(I)** (n ≥ 4). **(J, K)** Boxplot analysis of CVs of genes in and outside of CRRs\sub-CRRs\sub-CRRs with CRE30. Expressional coefficient of variations (CVs) of each gene was calculated using UCEC [**(J)** left], CESC[**(K)** left], and adjacent normal tissue [**(J, K)** right] data. Genes were divided into four groups: genes in sub-CRRs with CRE30, genes in sub-CRRs without CRE30, genes in CRRs (sub-CRR genes excluded), and genes in whole genome (all CRR genes excluded). The CVs of each group were box plotted. T-tests were performed, and significant differences were indicated by ** (p < 0.01).

To explore whether CRE30 had similar functions in other sub-CRRs, we selected sub-CRR96, which contains CRE30, in the endometrial tumor dataset for analyses. Nine genes are present in sub-CRR96, and the CRE30 segment is located in the second intron of the CYHR1 gene (**Figures S7A–C**). We compared gene expressions in this region between wild-type and CRE30 KO Ishikawa cells and found that gene upregulation in the CRE30 KO group was larger than that in wild-type cells (**Figure 6A** left), but there was no differences in the CYHR1 knockdown group (**Figure 6A** right). In response to SAHA, gene expression was significantly upregulated in wild-type cells, and the upregulation level was larger than that in the CRE30 KO group (**Figure 6B**). The results suggested that similar changes in gene regulation patterns were observed in CRE30 deletion and SAHA treatment in sub-CRR96.

ChIP experiments showed that CTCF bound to the CRE30 locus in sub-CRR96, and binding signals decreased after CRE30 deletion (**Figure 6C**). SAHA treatment (**Figure 6D**) and HDAC1/2 knockdown (**Figure 6E**) also resulted in decreased CTCF binding. These results indicate that CRE30 in sub-CRR96 recruits CTCF, and the binding events are affected by histone acetylation. Cell proliferation (**Figure 6F**) and migration (**Figure 6H**) were inhibited by CRE30 deletion in sub-CRR96, but no difference in cell activities was observed in the CYHR1 knockdown group in Ishikawa cells (**Figures 6G, I**).

Moreover, this 9-gene region was not identified as sub-CRR in the cervical cancer dataset (**Supplementary Table 2**), which implied that the CRE30 segment in the CYHR1 locus might not exhibit the same functions in cervical cancer. qPCR results in HeLa cells showed that the gene regulation pattern did not change in CRE30 KO cells (**Figure S7D**). ChIP assay revealed no CTCF binding signal on CRE30 (**Figure S7E**). Together, this indicates that in HeLa cells, CRE30 could not recruit CTCF binding or affect regional gene regulation.

Finally, we explored the effects of CRE30 at the genome level. By analyzing gene expression data of both cancer and adjacent normal tissues, we calculated the expression coefficient of variations (CVs) for each gene among different samples. Genes were divided into four groups: genes in sub-CRRs with CRE30, genes in sub-CRRs without CRE30, genes in CRRs (sub-CRR genes excluded), and genes in the whole genome (CRR genes excluded) (**Figures 6J, K**). The CVs of CRE30 sub-CRRs were significantly smaller than those of the CRRs or whole genome in both UCEC and CESC (p < 7e-6), indicating that the gene regulation range was obviously smaller in the presence of CRE30 (**Figures 6J** left, **6K** left), which is consistent with the CRE30-induced regulatory restriction identified above. The CVs of sub-CRR genes were mostly identical with those of the whole genome in normal tissues, indicating the marked difference of sub-CRR gene regulation patterns between cancer and normal tissues (**Figures 6J** right, **6K** right). Combined with the results showing cancer cell phenotype alterations induced by CRE30 deletion, these data supported that the gene regulation process mediated by CRE30 has biological significance in the process of carcinogenesis.

**Figure 7:**
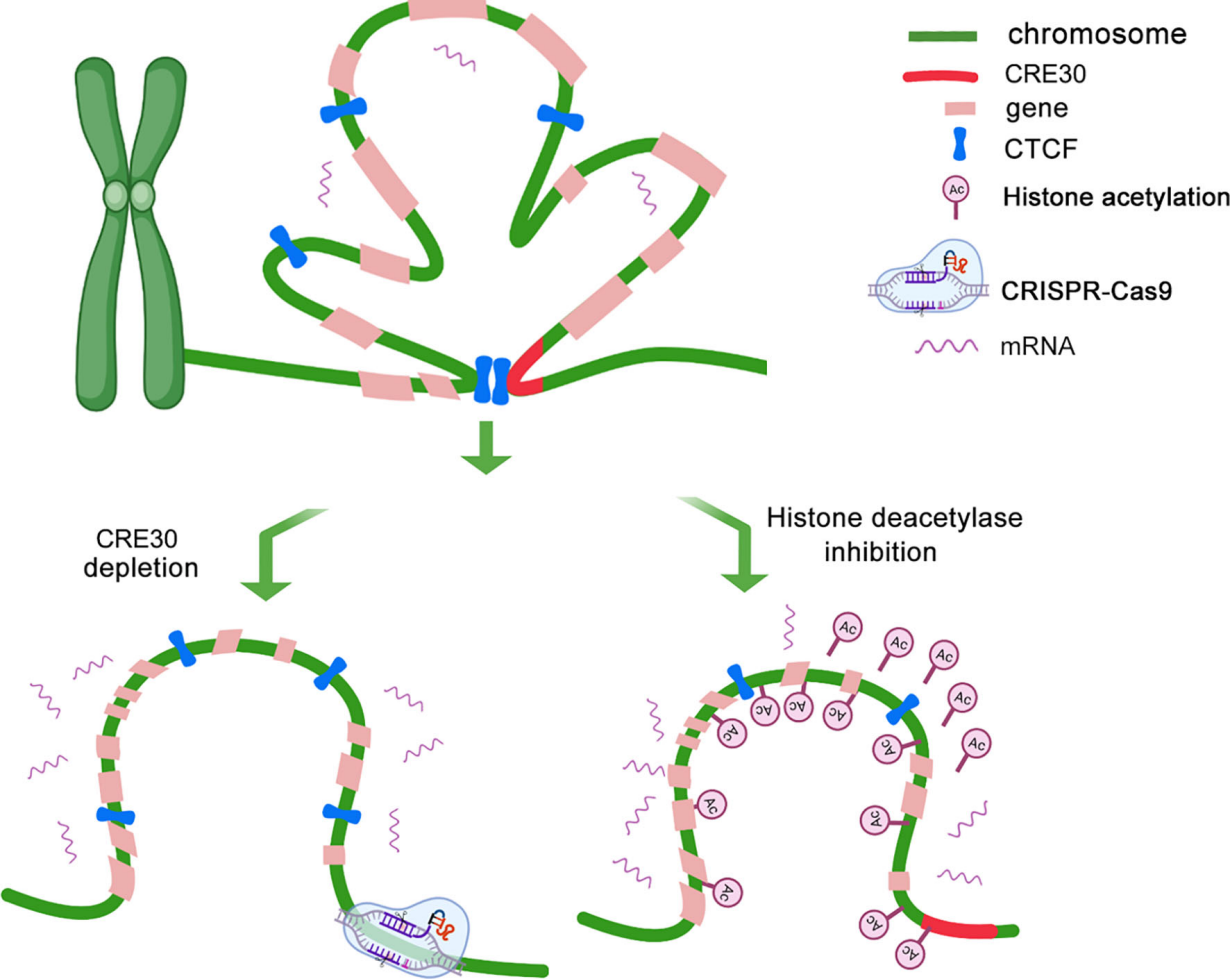
CRE30 collaborating with histone deacetylation maintains high-order chromatin organizations that affect gene expressions. A schematic for the potential model of expression regulation and chromatin architecture modulation in sub-CRR regions is shown. Top panel: in the sub-CRR region, CTCF is recruited on the CRE30 locus, and chromatin loops are maintained in sub-CRR regions, leading to compact 3D chromatin architecture, thereby limiting the gene regulation scope. Bottom left: CRE30 deletion abolishes CTCF binding, and chromatin loops are disrupted accompanied with gene regulation activation. In the bottom right model, increase in histone acetylation leads to disruption of CTCF binding on some sub-CRR locus and destruction of chromatin loops, which is further linked to active gene regulation.

Based on above findings, we developed a long-range gene regulation model based on the functional DNA element in cancer genomes (**Figure 7**). Under a low histone acetylation status, the conserved DNA element (CRE30) recruits CTCF and maintains chromatin loops in sub-CRRs, which leads to the restriction of gene expression regulation. With CRE30 deletion or histone acetylation modification, CTCF binding is disrupted and chromatin loops are destroyed, resulting in activation of gene regulation in sub-CRR containing CRE30.

## Discussion

Research on mechanisms for epigenetic remodeling including chromatin architecture and direct epigenetic modifications has confirmed their regulatory roles on long-range gene expression in specific regions of the cancer genome (3, 8, 28). However, little is known on the regulation patterns and functional elements. In this study, we classified interregional correlated sub-CRRs using a novel analysis pipeline and identified CRE30 as a functional element enriched within these regions. Deletion of CRE30 resulted in increased regional gene regulation and inhibited the proliferation and migration of tumor cells. We found that CRE30 contributed to establishing chromatin loops and maintaining compact chromatin architecture in sub-CRRs. We also observed that CTCF binding and chromatin loops on sub-CRR regions were disrupted by histone deacetylation inhibition, which further affected gene expression. We propose a model by which CRE30-maintained chromatin loops in sub-CRR regions restrict regional gene regulation and are modulated by histone acetylation.

Co-regulated regions have been reported in some different cancer genomes identified by different calculating methods (3, 8, 9). In this work, we identified CRRs using relative strict parameters. The spanning distance of CRRs (median distance from 0.22 to 0.3 Mb) was markedly shorter than co-expression regions in other studies (median distance from 0.9 to 1.86 Mb) (3, 8, 9) or the long-range epigenetic modification region (several megabases) (6, 26). The shorter DNA length greatly reduces the random occurrence of conserved DNA sequences, which facilitates further search for regulatory elements. Research in breast cancer observed interregional correlations between some of the regions (9). Here, we found for the first time that the distribution of correlations between CRRs is bimodal in many cancers, and we extracted sub-CRRs with a high interregional correlation. The sub-CRRs were hypothesized to share the same regulatory mechanisms, and we found that conserved CRE30 was enriched and demonstrated to affect regional expression patterns. Notably, two regions (sub-CRR95 and sub-CRR96) were predicted by our prediction algorithm to be affected by CRE30 in endometrial cancer, and both were verified in the Ishikawa cell line. In cervical cancer, only one region (sub-CRR95) was predicted to be included. Experimental results from HeLa cells confirmed that gene expression in the sub-CRR95 region was significantly affected by CRE30, but no difference was observed in the sub-CRR96 region. The results indicate that our method could accurately identify CRRs in different data sets and predict functional related elements.

In this study, the conserved DNA element CRE30 was found to recruit CTCF in different sub-CRR loci. The relationship between CTCF binding and chromatin architecture–induced gene regulation has been well established (40, 44). To gain mechanistic insights into CRE30 and long-range gene regulation, we first verified the recruitment of CTCF on CRE30 segments in different tumor types and loci using ChIP. Subsequent 3C and 3D-FISH assays showed the essential roles of CRE30 on long-range chromatin interaction and chromatin condensation. Our results again support the critical role of CTCF on long-range chromatin interactions and maintaining higher-order genome organization (19, 21, 45). Cohesin was considered to appear on CTCF-binding sites and contribute to establishment of chromatin loops (21, 39, 46). Here we found Rad21, a subunit of cohesion, to be also recruited on the CRE30 locus; the binding signals reduced with chromatin loop disruption that is induced by both CRE30 deletion and SAHA treatment. These results further indicated that CRE30 element contributed to maintaining of chromatin loops.

Previous studies suggested a weak influence of high-order chromatin structure on gene regulation in some loci in the absence of stress condition (46, 47). Ibrahim and Mundlos pointed out that rather than the steady state, tightly restricted regulatory events might be potential factors that help to describe other aspects of expressional effects (14). Moreover, sub-CRRs reflected a dynamic co-regulation process among clinical cases. We used serum starvation in short-term stress to perturb the transcriptome, and we found that gene upregulation was changed. Our work provided an alternative angle to describe the alteration of long-range gene expression and revealed the close relationship between high-order chromatin organization and dynamic long-range gene regulation patterns.

Long-range epigenetic remolding was found to associated with high-order chromatin alteration and gene regulation (41, 48). Recent research on histone acetylation revealed its function in facilitating chromatin interactions (43, 49). Here, by pharmacological and genetic targeting of the global histone deacetylation enzyme, we also observed chromatin loop alteration, which supported the functional roles of histone acetylation on three-dimensional genome modulation. However, different from the above works, our results showed that elevating histone acetylation in sub-CRR loci resulted in loop disruption and loss of chromatin locus proximity. This result indicated the complicacy and flexibility of epigenetic modifications in chromatin architecture modulation. Moreover, histone acetylation in other loci was found to regulate chromatin loops without changing CTCF binding (43, 49). In this research, we observed CTCF detaching in some loci in sub-CRRs; this might explain the different modulation directions of chromatin loops that are induced by histone acetylation here. This finding also provided another epigenetic modification that influences CTCF binding in specific loci besides DNA methylation (41, 42).

In our study, cancer case classification using gene expression diversity in sub-CRRs showed significant differences in patient survival time, which indicated that the regions that we identified might have practical values for clinical pathological feature prediction. Similar characteristics were also reported in co-expression regions in other studies (8, 9). Moreover, compared with using all sub-CRRs, survival analysis using sub-CRRs containing CRE30 showed significant differences in more tumor types. What is more, the proliferation and migration ability of cancer cells were reduced by CRE30 deletion. These observations strongly implied a critical role of CRE30-derived regulatory mechanisms on carcinogenesis. It also highlights the potential use of CRE30 in cancer survival prediction and classification.

Previous studies showed that the histone deacetylase inhibitor leads to epigenetic remodeling and chromatin architecture alteration of genomes (50). SAHA, a HDAC inhibitor, inhibits class I and II histone deacetylases and results in the accumulation of acetylated histones. This leads to the altered expression of important genes like antiproliferative and/or proapoptotic genes and impacts cell proliferation and differentiation (51). SAHA has been recommended as a new clinical antitumor drug in cutaneous T cell lymphoma, glioblastoma multiforme, and non-small lung carcinoma (52–54). Identifying targets of SAHA in cancer will help clarify its regulatory mechanism and improve its clinical application. In this study, we used SAHA for evaluating regional gene co-expression, chromatin architecture alteration, and CTCF binding. Our results showed that in the sub-CRR locus, histone acetylation induced by SAHA might regulate regional gene co-expression through affecting CTCF recruitment and altering the chromatin architecture. The findings provide new evidence for SAHA as a potential cancer therapy through regulating higher-order chromatin architecture.

In conclusion, our research developed a novel expressional correlation analysis pipeline to classified groups of co-regulation regions (defined as sub-CRRs here) exhibiting similar regulation patterns. A conserved DNA element (CRE30) was identified to enrich in sub-CRRs and proved to affect gene regulation and cancer cell proliferation and migration. CRE30 acts as an essential element to recruit CTCF and maintain chromatin loops, which restricts gene regulation in compact chromatin regions. Meanwhile, the regulatory restriction is relieved when histone acetylation disrupts CTCF binding and chromatin loops. Our results provide new findings into the collaboration among conserved DNA elements, epigenetic modification, and chromatin architecture on the long-range gene regulation process.

## Data Availability Statement

The original contributions presented in the study are included in the article/**Supplementary Material**. Further inquiries can be directed to the corresponding authors.

## Author Contributions

YuL and RT designed the research. RT performed the experiments. YiL and XL analyzed the data. FH analyzed the data and drew the figures and tables. ZL performed experiments on sub-CRR96. HS and XZ performed some 3D FISH experiments and supplementary data. HN and QJ performed the writing and reviewing of the manuscript. All authors contributed to the article and approved the submitted version.

## Funding

This work was supported by the project from the National Natural Science Foundation of China (no. 31571323 to YuL, no. 62032007 to QJ, and no. 62002086 to YiL), Heilongjiang Touyan Team (HITTY-20190034), and Heilongjiang Postdoctoral Science Foundation funded project (LBH-Z20064). 

## Conflict of Interest

The authors declare that the research was conducted in the absence of any commercial or financial relationships that could be construed as a potential conflict of interest.

## Publisher’s Note

All claims expressed in this article are solely those of the authors and do not necessarily represent those of their affiliated organizations, or those of the publisher, the editors and the reviewers. Any product that may be evaluated in this article, or claim that may be made by its manufacturer, is not guaranteed or endorsed by the publisher.
