# Supplementary material for: A CTCF-Binding Element and Histone Deacetylation Cooperatively Maintain Chromatin Loops, Linking to Long-Range Gene Regulation in Cancer Genomes
Source: Front Oncol. 2022 Jan 21;11:821495. doi: 10.3389/fonc.2021.821495 (PMC8813737; doi:10.3389/fonc.2021.821495)
Supplement: Supplementary file 6 [file DataSheet_1.pdf]

Supplementary Table 1 **Oligo sequences that used in this research**
Supplementary Table 2 **Coordinately regulated regions raw data**
Supplementary Table 3 **CRE30 sequences in *HSF1* locus**
Supplementary Table 4 **CRE30 binding protein candidates**
Supplementary Table 5 **Enriched GO items of CRE binding protein candidates**

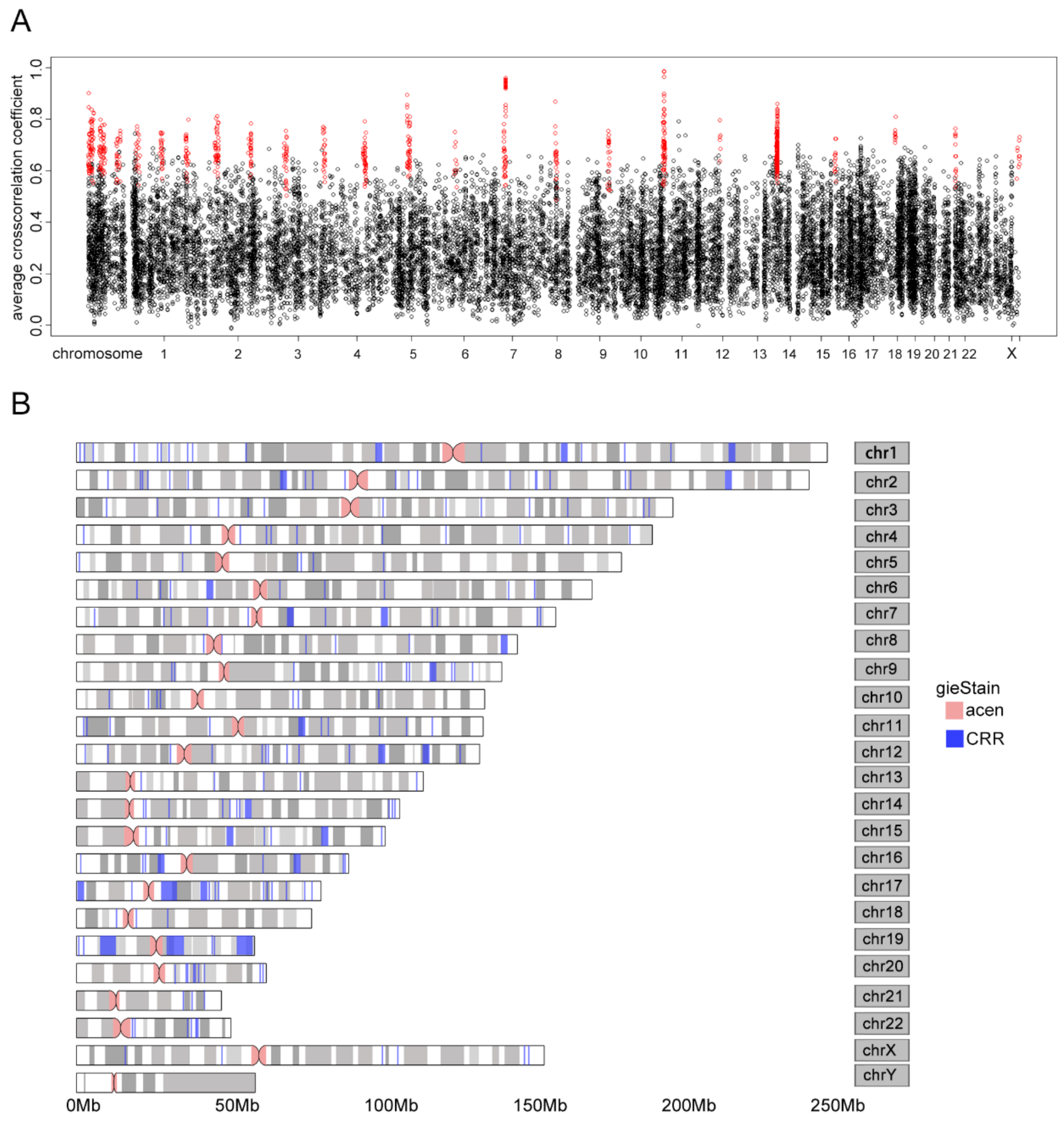

Figure S1

CRRs with high expressional intra-region correlations existed in the whole cancer genome. **A** Expressional correlation coefficient among every 5 adjacent genes was calculated using UCEC data from TCGA, the average value of each unit was mapped on to chromosomes. Red circles marked units included in CRRs. **B** Spanning loci of CRRs in different chromosomes were displayed and marked as blue.

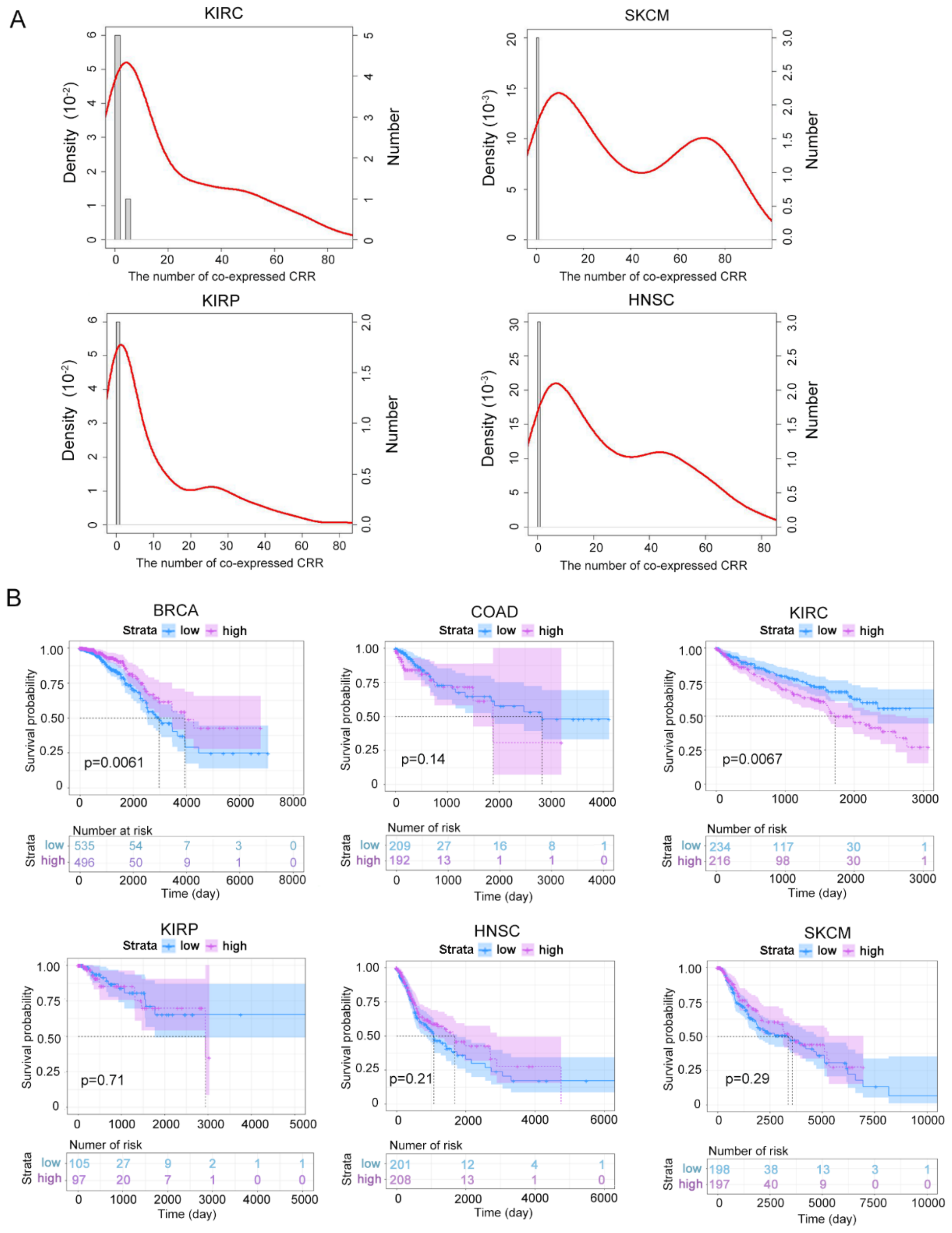

Figure S2

Sub-CRRs showed high expressional inter-region correlations and associated with cancer survival time. **A** Distribution of CRRs which have significant correlation ($p<0.05$) with other CRRs. Horizontal coordinates indicated quantities of "other CRRs" that correlated with one given CRR, the left vertical coordinate counted the proportion of such "given CRRs". Proportions of CRRs from different cancer data were plotted on the red curves. Take KIRC as an example, a point (x, y) on the red curve indicate a group of CRRs, each of them has significant correlation with x (the quantity) other CRRs, and the proportion of such group of CRRs compared with the whole is $y\times10^{-2}$.

Quantity distribution (right ordinate) obtained with randomized CRRs of the same size was showed as the gray bars. Right peaks indicated high inter-region correlation among sub-CRRs. B Survival curves of different clinical case groups that classified by sub-CRR genes showed significant difference in BRCA and KIRC.

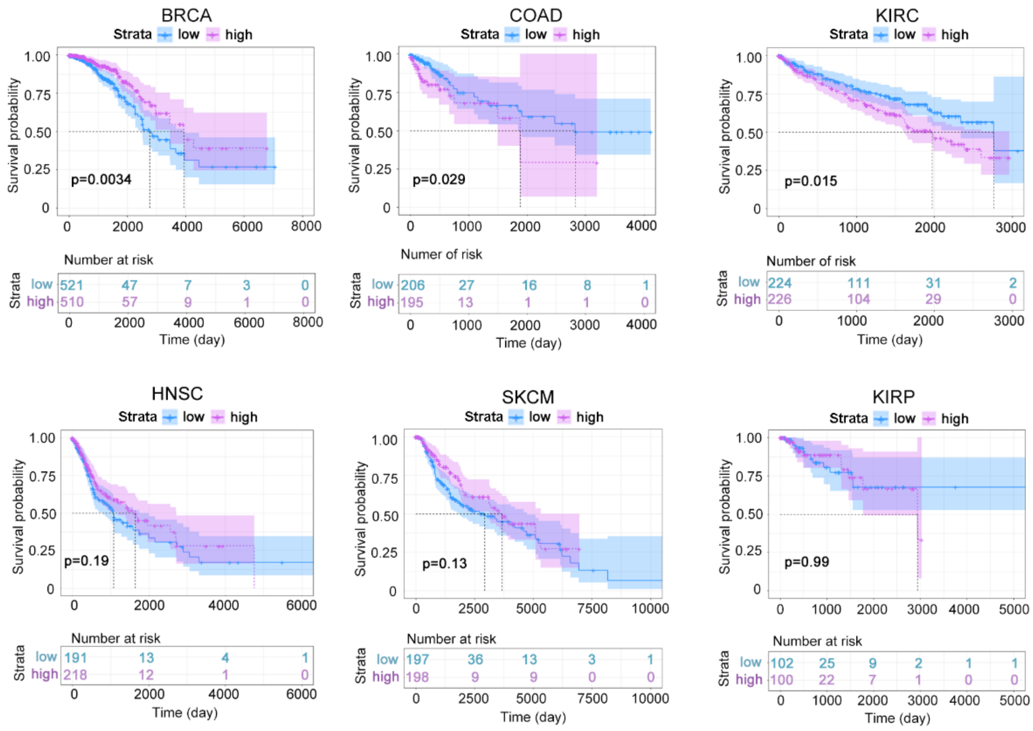

Figure S3

CRE30 contained sub-CRRs were associated with cancer survival. Survival curves of different clinical case groups that classified by genes in CRE30 contained sub-CRRs. Obvious differences were observed in BRCA, COAD and KIRC.

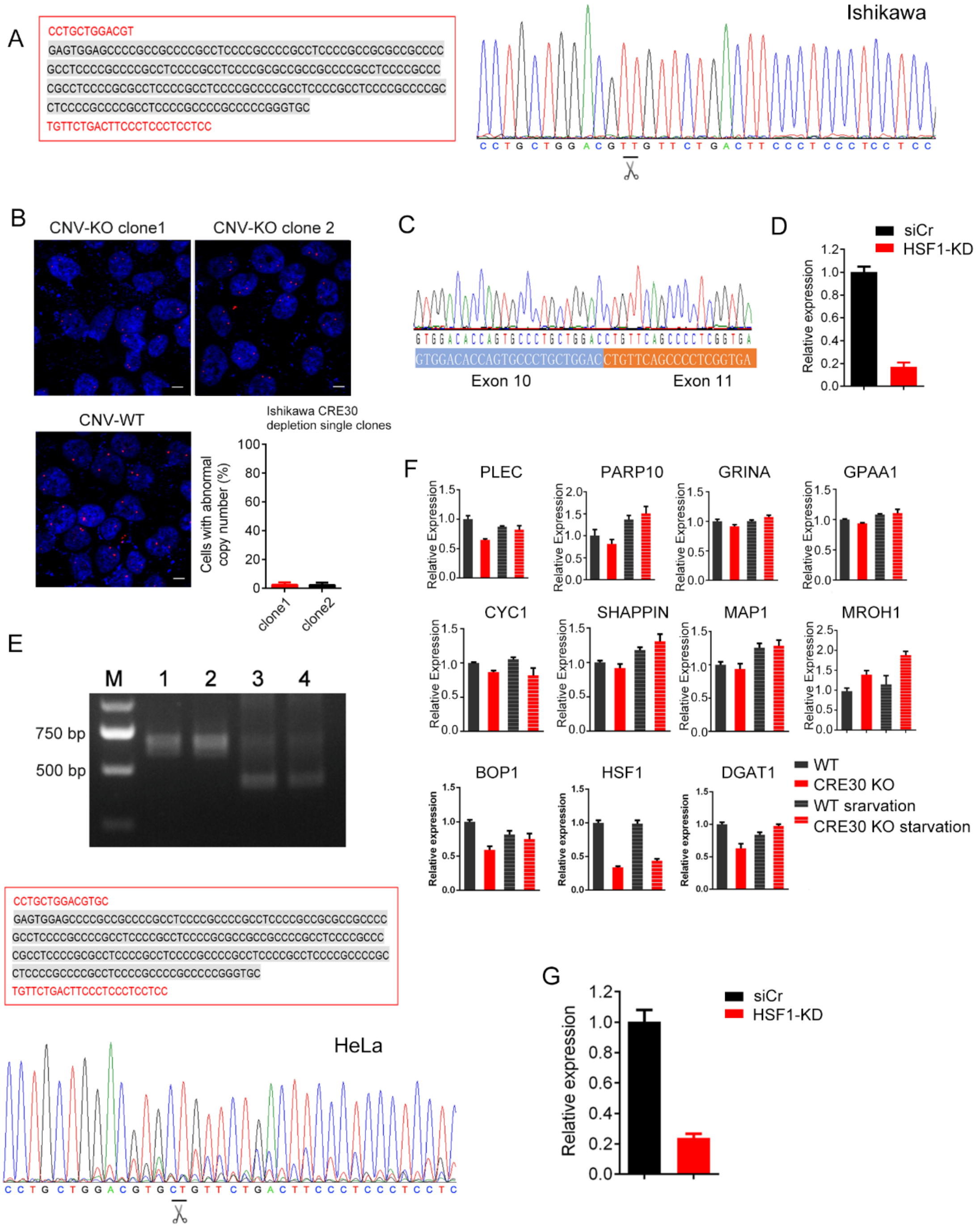

Figure S4

CRE30 deletion in Ishikawa and HeLa cells. **A** Nuclear acid bases showed cutting range by CRISPR/Cas9 in Ishikawa cells, CRE30 sequences that knocked out was labeled with gray. The chromatogram showed sequences around cutting site (marked with scissor) in deletion cells. **B** FISH experiments was used to detect copy number variants in wild type and CRE30 deletion clones of Ishikawa cells. FISH probe (red) that spanning HSF1 regions was used, and nucleus labeled with DAPI (blue). Representative FISH images were showed, scale bars on bottom right of each photo indicated 10 μm. Percentages of nucleus with abnormal copy number were showed with columns, which confirmed that similar with wild type cells, copy number of HSF1 locus

had no obvious change in CRE30 deletion cells. **C** Chromatogram indicated cDNA sequence around splicing site between HSF1exon 10 and 11, bases on the bottom showed the sequences of exon 10 (blue) and 11 (orange) respectively. **D** *HSF1* siRNA knock down efficiency was determined by qPCR in Ishikawa cells. **E** Photo of agarose electrophoresis illustrated verification PCR bands for wild type and deletion HeLa cells (upper panel). Wild type HeLa cells (lane 1, 2) had one band around 600bp, the mixed deletion clones (lane 3, 4) had both of the wild type band and deletion band (shorter than 500bp). Lower panel showed cutting range by CRISPR/Cas9 in Ishikawa cells, element sequences that knocked out was labeled with gray. The chromatogram showed sequences around cutting site (marked with scissor) in deletion cells. **F** qPCR analysis for HeLa cells sub-CRR 95 genes expression following deletion of CRE30 and serum starvation. Black columns indicated wild type cells, red columns indicated CRE30 KO cells, columns with horizontal stripe showed expression pattern after serum-starvation. **G** SiRNA knock down efficiency was determined by qPCR in HeLa cells, which indicated about 75% RNA level decreasing efficiency.

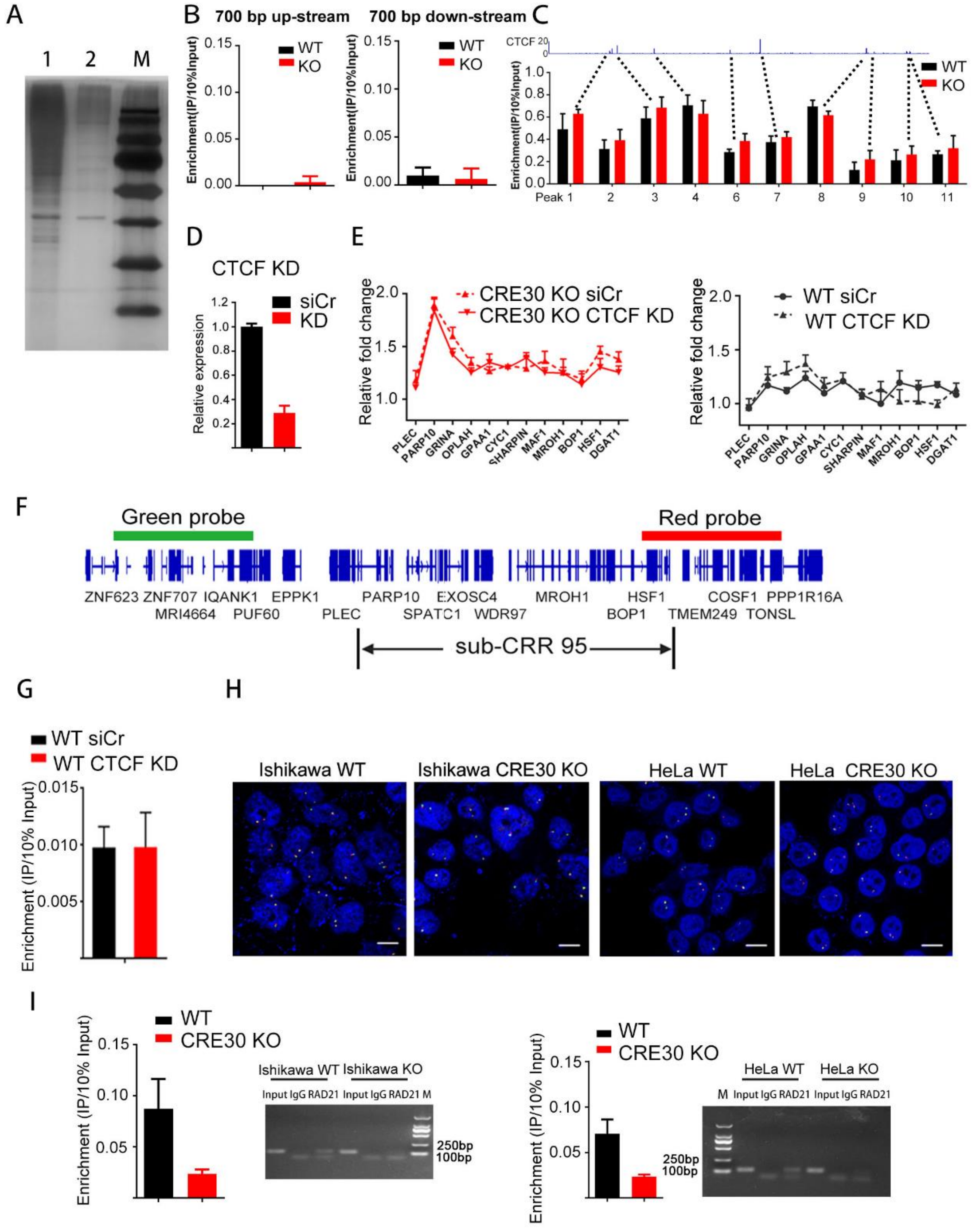

Figure S5

CRE30 was associated with chromatin architecture maintaining. **A** Proteins obtained from DNA pull-down experiments were detected by SDS-PAGE following with silver stain. Samples from 300bp probe (lane 1), random control probe (lane 2) were shown with protein marker (lane M). **B** CTCF ChIP-qPCR of CRE30 up and down stream control locus. Primers were designed 700 bp upstream and 750 bp downstream of CRE30 (Table S1), then the CTCF binding signal was detected in wild type and CRE 30 KO cells by qPCR. **C** ChIP-qPCR assayed other CTCF binding sites in sub-CRR95. Binding loci detected based on ChIP-seq data from Ishikawa (ENCFF961BQG, ENCODE). Peak number corresponds to primers listed in Table S1 **D** QPCR assay

confirmed about 70% of CTCF knock-down efficiency in Ishikawa cells. **E** Line charts showed expressional fold change of genes before (left panel) and after (right panel) CTCF siRNA knocking down (n=3). **F** ChIP-qPCR columns chart measuring CTCF binding frequency on CRE30 locus before and after CTCF knocking-down (n=2). **G** Schematic presentation of probe position that used in 3D-FISH experiments. Green and red fluoresce probes were designed close to up and down-stream boundaries of sub-CRR 95. **H** Representative 3D-FISH images of Ishikawa cells and Hela cells before and after CRE30 deletion. Boundary loci of sub-CRR (red and green) and DAPI (blue) are shown. Scale bars indicated 10 μm. **I** qPCR (left) and PCR (right) were performed to detect Rad21 binding in wild-type and CRE30 deletion Ishikawa/HeLa cell lines (n=3).

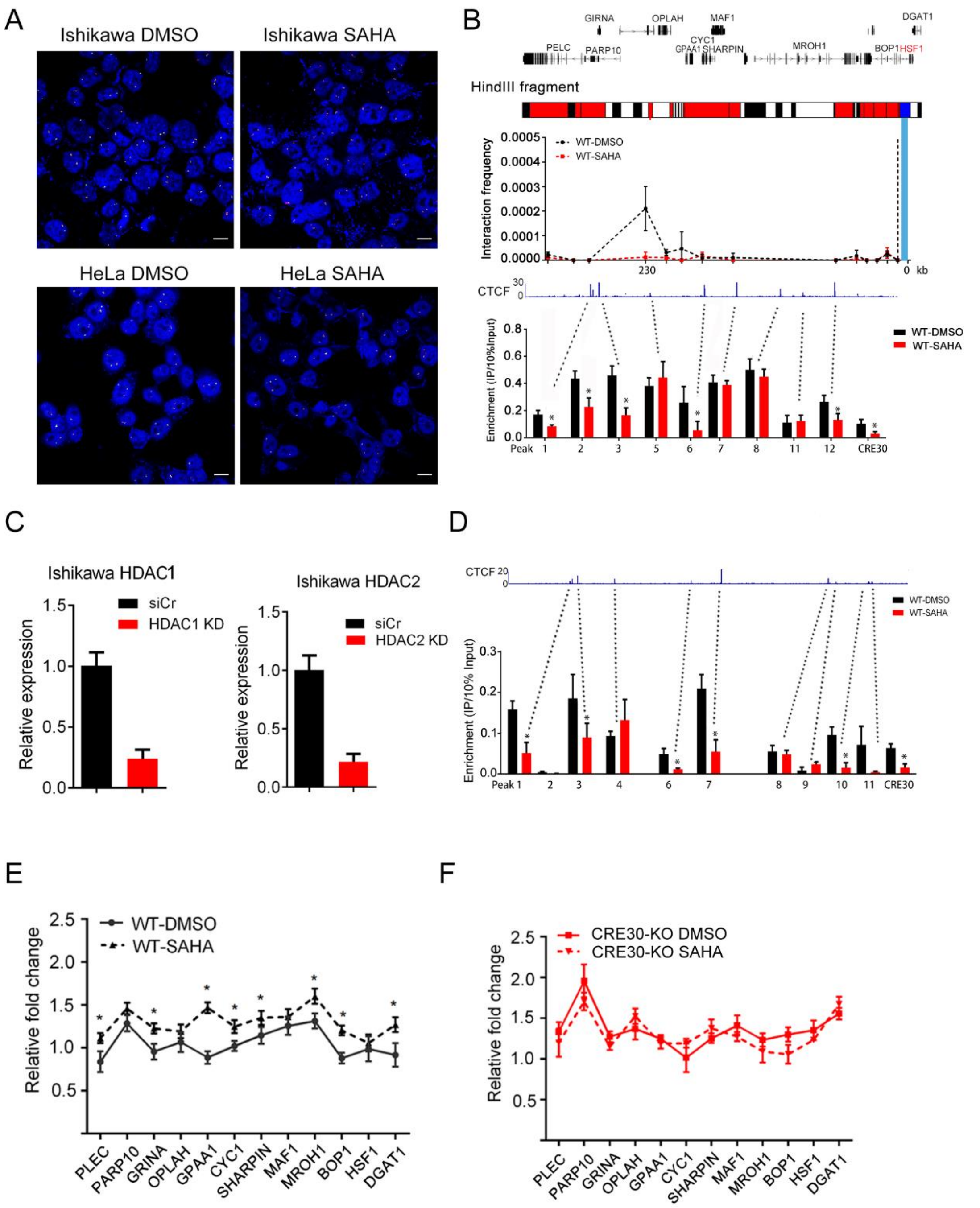

Figure S6

Histone acetylation affected chromatin loops and regional gene regulation in sub-CRR 95. **A** Representative 3D-FISH images of Ishikawa cells (upper panel) and HeLa cells (lower panel) with DMSO or SAHA treatment. Boundary loci of sub-CRR (red and green) and DAPI (blue) are shown. White scale bars indicated 10 μm. **B** HeLa cell 3C-qPCR at sub-CRR 95 region (upper panel). The hind III fragments bars on the top illustrate bait fragment (blue) and tested interaction fragments (red). Interaction peaks were detected in in DSMO group (black dotted lines), which decreased significantly with SAHA treatment (red dotted lines). Lower panel showed CTCF ChIP-qPCR results before (black columns) and after (red columns) SAHA treatment. Binding loci detected

based on ChIP-seq data from HeLa cells[49]. Peak number corresponds to primers listed in Table S1. **C** QPCR determined siRNA knock-down efficiency about 70% for both HDAC1 and HDAC2 in Ishikawa cells. **D** ChIP-qPCR detected Rad21 binding signal changes before (black columns) and after (red columns) SAHA treatment in sub-CRR 95 locus. **E** and **F** QPCR experiments were performed on HeLa cells following with SAHA treatment, gene up-regulation scope increased widely in wild type group (E) on both conditions, but only had very few or modest change in element deletion cells (F).

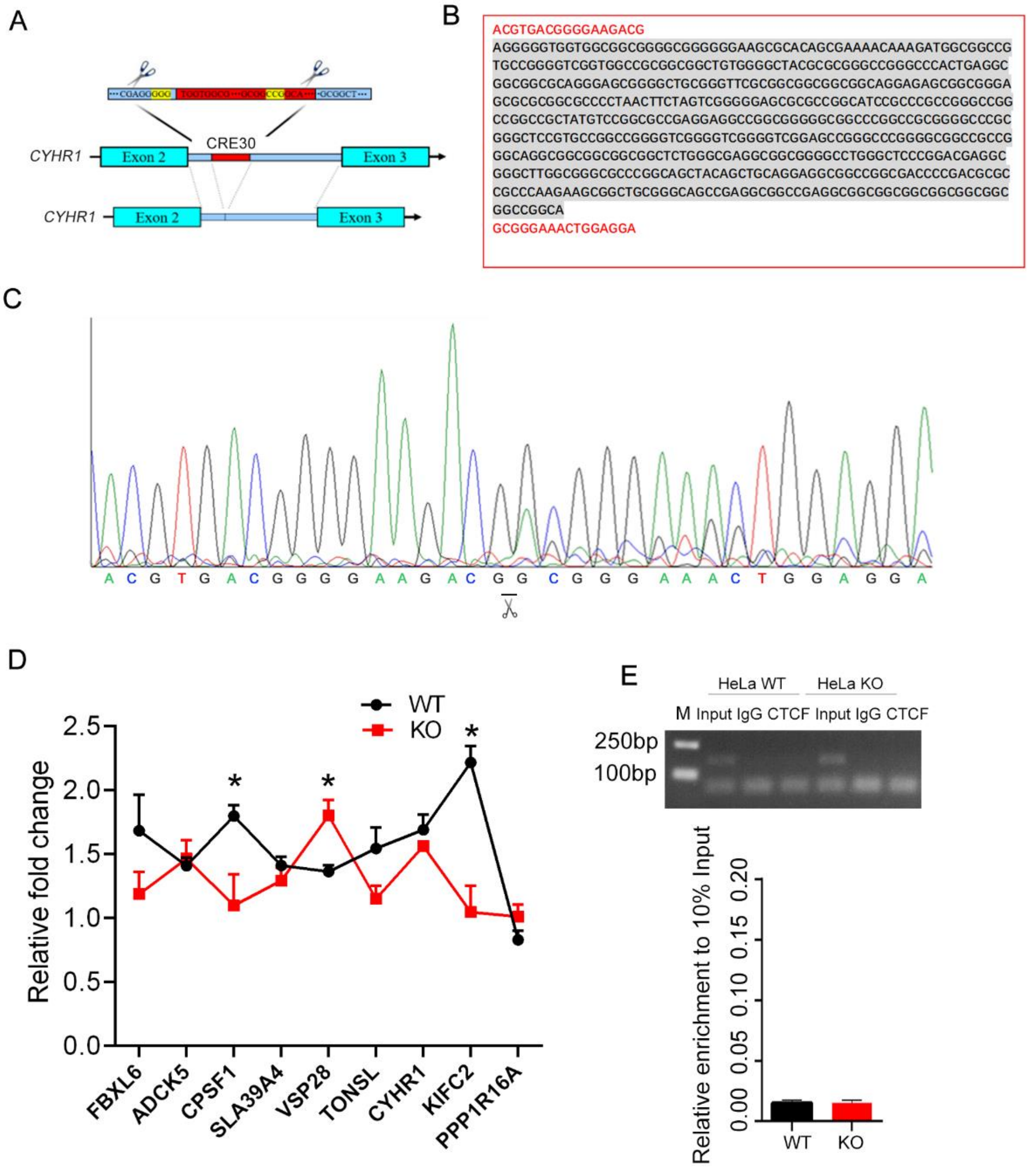

Figure S7

Sub-CRR 96 CRE30 deletion. **A** Schematically showed CRE30 location and CRISPR deletion process in *CYHR1* gene region, red rectangle represented CRE30 locus, PAM sequences that used for CRISPR were lightened with yellow. **B** Nuclear acid bases showed cutting range by CRISPR/Cas9 in Ishikawa cells, CRE30 sequences in *CYHR1* locus that knocked out was labeled with gray. **C** The chromatogram showed sequences around cutting site (marked with scissor) in deletion cells. **D** Relative change fold of sub-CRR 96 gene expression under serum-starvation stress in HeLa wild type (black) and CRE30 deletion (red) cells. Results showed that there was no widely increasing regulation scope observed in HeLa CRE30 deletion cells. E ChIP-PCR results didn't observe CTCF binding signal on sub-CRR 96 CRE30 locus in HeLa cells.
